# The High–Low Arctic boundary: How is it determined and where is it located?

**DOI:** 10.1002/ece3.10545

**Published:** 2023-09-28

**Authors:** Ksenia A. Ermokhina, Anna I. Terskaia, Tatiana Yu. Ivleva, Sergey V. Dudov, Vitalii А. Zemlianskii, Michael Yu. Telyatnikov, Olga V. Khitun, Elena I. Troeva, Natalia E. Koroleva, Svetlana Yu. Abdulmanova

**Affiliations:** ^1^ A.N. Severtsov Institute of Ecology and Evolution Russian Academy of Sciences Moscow Russia; ^2^ Faculty of Space Research Lomonosov Moscow State University Moscow Russia; ^3^ Department of Physical Geography and Ecosystem Science Lund University Lund Sweden; ^4^ Faculty of Biology Lomonosov Moscow State University Moscow Russia; ^5^ University of Zurich Department of Evolutionary Biology and Environmental Studies Zurich Switzerland; ^6^ Central Siberian Botanical Garden SB of Russian Academy of Sciences Novosibirsk Russia; ^7^ Komarov Botanical Institute Russian Academy of Sciences St.‐Petersburg Russia; ^8^ Institute for Biological Problems of Cryolithozone SB of Russian Academy of Sciences Yakutsk Russia; ^9^ Polar‐Alpine Botanical Garden‐Institute Kola Scientific Centre of the Russian Academy of Sciences Kirovsk Russia; ^10^ Institute of Plant and Animal Ecology Ural Branch of Russian Academy of Sciences Arctic research Station Labytnangi Russia

**Keywords:** Arctic Vegetation Archive, biogeography and macroecology, ERA5, geobotanical boundaries, MODIS, plant–climate interactions, remote sensing data (Landsat), species distribution modeling (SDM), the High and Low Arctic, Western Siberia

## Abstract

Geobotanical subdivision of landcover is a baseline for many studies. The High–Low Arctic boundary is considered to be of fundamental natural importance. The wide application of different delimitation schemes in various ecological studies and climatic scenarios raises the following questions: (i) What are the common criteria to define the High and Low Arctic? (ii) Could human impact significantly change the distribution of the delimitation criteria? (iii) Is the widely accepted temperature criterion still relevant given ongoing climate change? and (iv) Could we locate the High–Low Arctic boundary by mapping these criteria derived from modern open remote sensing and climatic data? Researchers rely on common criteria for geobotanical delimitation of the Arctic. Unified circumpolar criteria are based on the structure of vegetation cover and climate, while regional specifics are reflected in the floral composition. However, the published delimitation schemes vary greatly. The disagreement in the location of geobotanical boundaries across the studies manifests in poorly comparable results. While maintaining the common principles of geobotanical subdivision, we derived the boundary between the High and Low Arctic using the most up‐to‐date field data and modern techniques: species distribution modeling, radar, thermal and optical satellite imagery processing, and climatic data analysis. The position of the High–Low Arctic boundary in Western Siberia was clarified and mapped. The new boundary is located 50–100 km further north compared to all the previously presented ones. Long‐term anthropogenic press contributes to a change in the vegetation structure but does not noticeably affect key species ranges. A previously specified climatic criterion for the High–Low Arctic boundary accepted in scientific literature has not coincided with the boundary in Western Siberia for over 70 years. The High–Low Arctic boundary is distinctly reflected in biodiversity distribution. The presented approach is appropriate for accurate mapping of the High–Low Arctic boundary in the circumpolar extent.

## Graphical abstract

This study focuses on geobotanical subdivision of the Arctic and provides insight into the structure of vegetation cover, biodiversity and functional traits distribution, and their spatial relations. It is shown that the High–Low Arctic boundary is distinctly reflected in biodiversity distribution and that the widely accepted climatic criterion used in delimitation schemes is not reliable for a huge part of the Arctic biome.

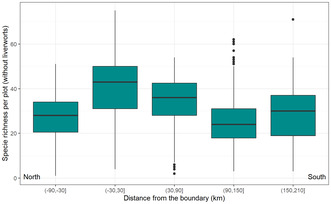

## 1 INTRODUCTION

Geobotanical subdivisions, such as vegetation zones and subzones, are of paramount importance in ecological research, facilitating the comprehension of ecological patterns and processes across diverse spatial scales. These subdivisions establish a framework for the classification of vegetation types, enabling comparisons between ecosystems, investigations into their dynamics, and assessments of their responses to environmental changes. Geobotanical subdivision finds common applications in the analysis of climate change impacts on vegetation, the assessment of biodiversity patterns and conservation priorities, the study of landscape‐level ecological processes, and the investigation of ecosystem functioning. The specific applications and research findings associated with geobotanical subdivision may vary depending on the region, research focus, and employed methods.

Accurate geobotanical delimitation is essential for both fundamental scientific research and applied ecological studies, as each subzone exhibits distinct vegetation characteristics related to community composition, species diversity, biomass, structure, density, and ecosystem features. These characteristics influence various aspects, including snow distribution, albedo, and soil properties, making precise delimitation crucial. The revisitation of geobotanical boundaries acknowledges the dynamic nature of ecosystems, facilitates adaptation to environmental changes, and supports more effective conservation practices. Regular reassessment and updating of boundaries refine our understanding of vegetation patterns, ensuring its relevance and providing a robust foundation for ecological research and management.

General principles of geobotanical subdivision are built on features of vegetation structure and floral composition that depend on the hierarchical level of delimitation, and on climate peculiarities. Despite common principles, there are various delimitation schemes existing even at the top hierarchical (planetary) level, and there are plenty of studies based on them. The difference in the schemes of geobotanical subdivision used in various ecological studies leads to limitations in results comparison. The effect becomes stronger at the levels of zonal and subzonal division. Also, this is especially pronounced at the margins of life, where the environmental situation changes sharply over short distances, and where the structure of vegetation cover controls most aspects of ecosystems' functioning such as carbon cycle, biodiversity, and stress resistance.

The zonal position of an area and its associated ecosystem characteristics are important basic information for Arctic researchers. The majority of work devoted to geobotanical subdivision of the tundra zone was written in the middle of the last century and was based on scanty field data (Alexandrova, 1980; Gorodkov, 1935; Lavrenko & Sochava, 1954; etc.). The subzone borders were drawn based on speculative extrapolation of temperature data. This had obvious limitations for some Arctic regions as there were only a few meteorological stations on the coast. Changes in climatic conditions during the last decades may have moved the zonal and subzonal tundra borders to the north. New field and remote sensing (RS) data show that significant changes have taken place in the arctic vegetation cover (Elmendorf et al., 2012; Epstein et al., 2017; Myers‐Smith et al., 2020). However, the recently created maps follow old subdivision schemes (e.g., Ogureeva et al., 2018; Raynolds et al., 2019).

**FIGURE 1:**
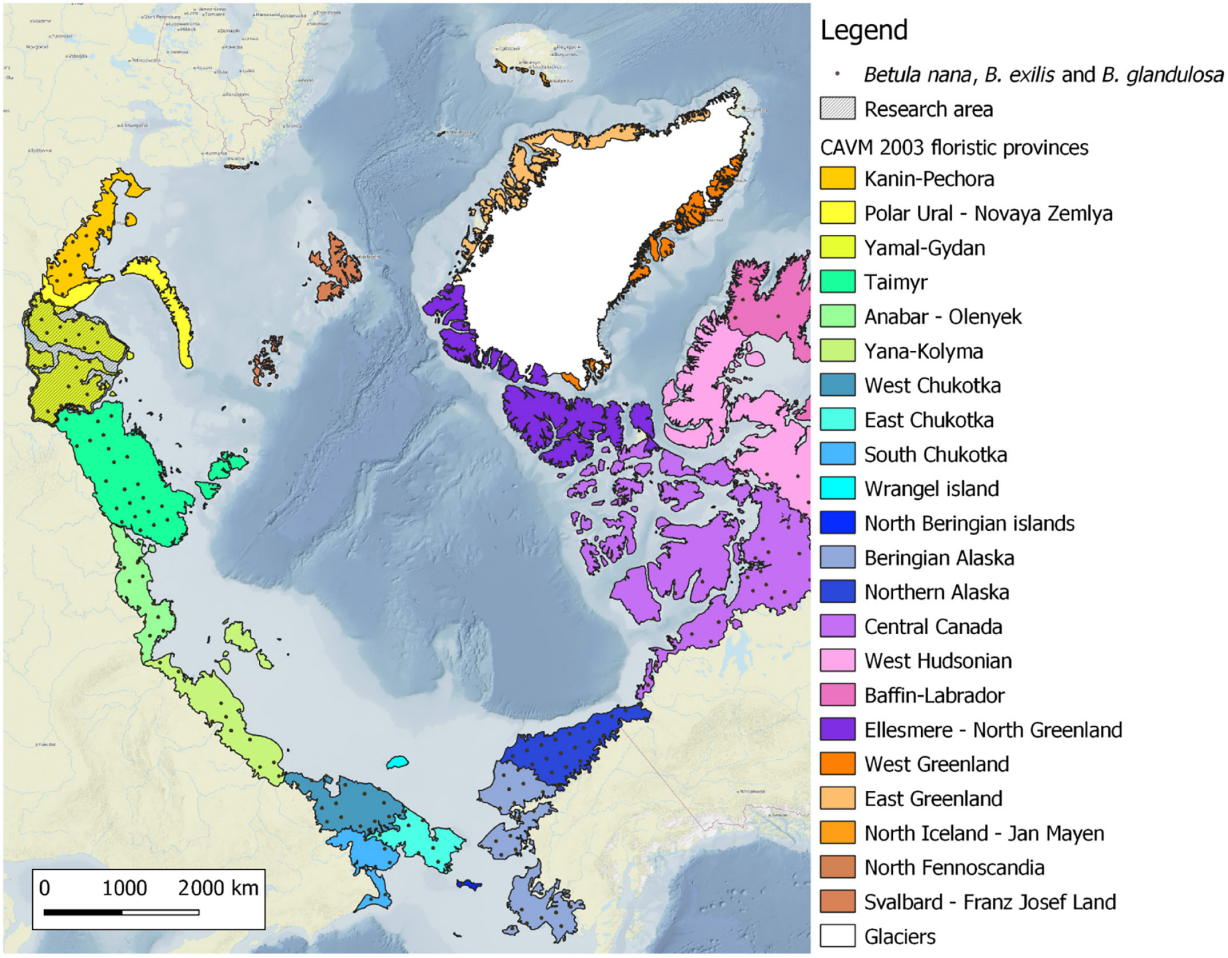
Overview map of the Arctic. The floristic subdivision is displayed following the Circumpolar Arctic Vegetation Map (CAVM Team, 2003) with modifications provided in Zemlianskii et al., 2023. The joint circumpolar distribution of dwarf birches *Betula nana*, *B. exilis*, and *B. glandulosa*, which are the main shrub species used for geobotanical delimitation of the High and Low Arctic, is shown based on data from the Global Biodiversity Information Facility (GBIF, https://www.gbif.org). Anthropogenic habitats of these species were filtered using intact/modified categories from the global record of annual terrestrial Human Footprint dataset from 2000 to 2018 (Mu et al., 2022); natural habitats were aggregated by 100 × 100 km grid and shown on the overview map.

The terms “Arctic,” “tundra zone,” and “tundra” are closely related but have distinct nuances. The Arctic refers to the northernmost region of the Earth (Figure 1), encompassing geographical, climatic, and biological aspects. The Arctic covers approximately 8% of the global land surface north of the boreal forest tree line (Alexandrova, 1980; Bliss & Matveyeva, 1992; McGuire et al., 1997; Raynolds et al., 2008; Walker et al., 2005). The tundra zone specifically pertains to the vegetation cover of the Arctic. Tundra represents a vegetation type dominated by dwarf shrubs, perennial herbs, mosses, and lichens, which is most common within the tundra zone. In the article, we address the distinctive characteristics of High and Low Arctic communities, as recognized in scientific literature. In geobotany, the concept of “vegetation cover” encompasses two components: “flora,” which refers to the historically established combination of species within a given area, including both species that are indicative of present conditions and relics from previous periods, and “vegetation,” which represents the assemblages of plants (communities) typical for that specific area and reflecting the current abiotic conditions. To simplify, the two aspects of vegetation cover are as follows: (1) taxonomical, which is connected to the flora, and (2) phytocoenotic, which is connected to the vegetation. While we use the term “boundary” in cartographic traditional application, it is important to note that it represents more than just a line; it signifies a transition, a kind of “corridor” between zones or subzones. A concise glossary of geobotanical terminology used throughout the article is presented in Appendix S1.

Detailed interpretation of the correspondence between Soviet geobotanists' delimitations of the Arctic (Alexandrova, 1980; Matveeva, 1998) and bioclimatic zones of the Circumpolar Arctic Vegetation Map (CAVM) was presented by Walker et al. (2005). On the CAVM, subzones B and C of the High Arctic correspond to two stripes (southern and northern) of the subregion of arctic tundra, subzones D and E of the Low Arctic – to northern and southern stripes of the subarctic tundra subregion (Alexandrova, 1980). The border between the arctic and subarctic tundra (subzones C and D of the CAVM), arctic and northern hypoarctic tundra subzones of Yurtsev (1994), or arctic and typical tundra of Gorodkov (1935) has been recognized for its great geographical and botanical importance. Alexandrova (1980) considered it a boundary between “two subregions of the tundra region”. Sochava (1952) and Yurtsev (1966) gave it an even higher rank since it divides arctic and hypoarctic botanical–geographical belts. In American phytogeographical literature, it is the border between the High and Low Arctic (Bliss, 1997; Epstein et al., 2004) and it is also recognized as the most dramatic shift in ecosystem properties compared to any other transitions that occur within the Arctic (Epstein et al., 2004).

This boundary is defined by the change in the dominant plant functional groups in zonal communities and the ratio of phytogeographical groups in the flora (Alexandrova, 1980; Elvebakk, 1999). In the High Arctic subzone, synusia of arctic and arctic–alpine prostrate dwarf shrubs become dominant in zonal communities, while hypoarctic shrubs and dwarf shrubs disappear. In the High Arctic, the flora loses its southern elements (boreal and hypoarctic species), and the proportion of different geographical groups in the flora changes from almost equal participation to absolute predominance of the arctic group (Alexandrova, 1980; Khitun et al., 2007, 2016; Rebristaya, 2013; Yurtsev, 1994). Yurtsev (1966) wrote about fundamentally different origins of the flora of these subzones. The difference between the High and Low Arctic is strengthened by the fact that, according to the paleobotanical data, the area occupied by the High Arctic tundra remained treeless during the whole Pleistocene epoch (Giterman et al., 1968; Velichko et al., 2002), whereas the Low Arctic experienced taiga expansion during warmer periods. It resulted in the enrichment of its flora with boreal species and affected the communities' structure and composition.

We focus our study on the West Siberian Arctic, one of the Arctic floristic provinces (Yamal‐Gydan province, Figure 1), where no special research has ever been carried out to determine the exact position of geobotanical boundaries. The main difficulty in mapping the boundary's position is the shortage of field studies and the uneven distribution of those that do exist (refer to Appendix S1 for a concise overview of the research conducted in the West Siberian Arctic prior to the current study). The mismatch in position of subzonal borders on the widely accepted schemes of delimitation of the Arctic (Alexandrova, 1980; Gorodkov, 1935; Yurtsev, 1994) and field observations on the Gydansky Peninsula was noted earlier (Khitun, 2003; Khitun et al., 2007; Rebristaya & Khitun, 1994). However, the small number of sites studied did not allow the determination of a final conclusion. We carried out field geobotanical research (mainly in 2017), then standardized and analyzed field material to characterize the present‐day vegetation cover of the West Siberian Arctic (Telyatnikov et al., 2019a, 2019b, 2021a, 2021b; AVA, https://avarus.space) and to clarify the location of the High–Low Arctic boundary. Our approach to define the position of the High–Low Arctic boundary is based on the established properties of Arctic subzonal communities and utilizes traditional criteria along with modern remote sensing, climatic, and field data. The main objectives of the article are as follows:

1. Analyzing different delimitation schemes for the High–Low Arctic boundary and identifying common criteria.
2. Investigating the impact of human activities on the distribution of biotic delimitation criteria.
3. Assessing the relevance of the widely accepted temperature criterion in light of ongoing climate change.
4. Developing an accurate mapping approach for the High–Low Arctic boundary using vegetation, remote sensing, and climatic data.
5. Determining the position of the High–Low Arctic boundary in Western Siberia.

## 2 STUDY AREA

The West Siberian Arctic covers a land surface of approximately 300,000 km^2^ and encompasses the peninsulas of Yamal, Gydansky, and Tazovsky. In the following Sections 2.1, 2.2, 2.3, 2.4, 2.5–2.5), we provide a brief description of the natural features of the West Siberian Arctic for better contextualization of our study.

**FIGURE 2:**
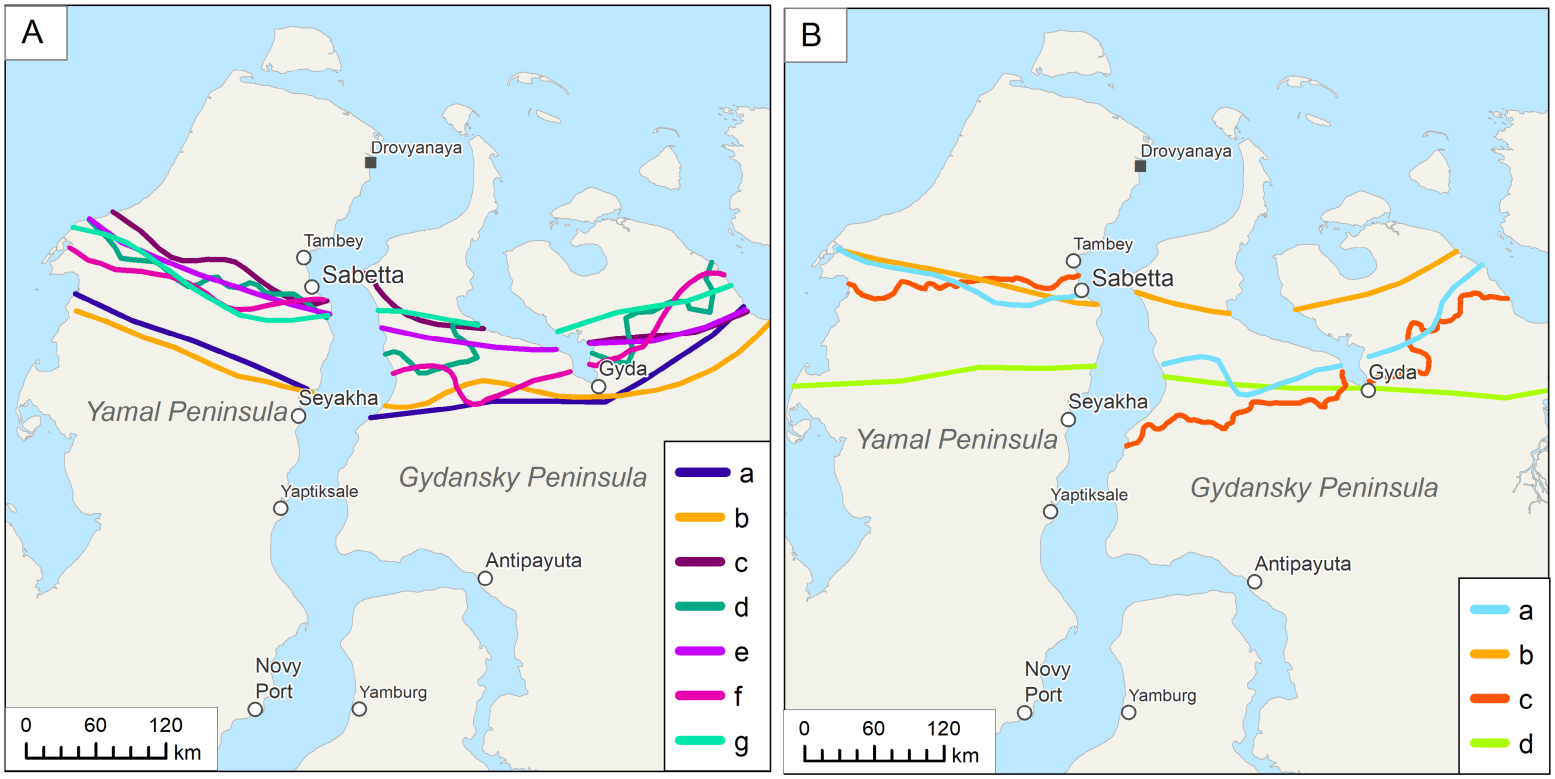
The position of the High–Low Arctic boundary in different delimitation schemes. (A) The High–Low Arctic boundary in the geobotanical studies: a, the border of the arctic tundra subzone (Gorodkov, 1935); b, first‐order belt border of the arctic tundra region (Map of geobotanical subdivision of the USSR, 1947); c, the arctic tundra border (Lavrenko & Sochava, 1954); d, the border of the arctic tundra subzone (Ilyina et al., 1976); e, the border of the arctic tundra subzone (Alexandrova, 1980); f, the southern border of the arctic tundra (map “Zones and types of vegetation in Russia”; Ogureeva et al., 1999); g, the southern border of the arctic tundra subzone (Yurkovskaya & Safronova, 2019). (B) The High–Low Arctic boundary in thematically related works: a, the border of the High Arctic biome (Ogureeva et al., 2018); b, the southern border of botanical geographical subzone of the arctic tundra (Khitun, 2005); c, the border of subzone C (Circumpolar Arctic Vegetation Map; CAVM Team, 2003); d, the northern border of the hypoarctic botanical geographical belt (Yurtsev, 1966).

We analyzed the location of the boundary between the High and Low Arctic in Western Siberia using the schemes of different authors (Figure 2). All the borders reflect a dramatic change in the structure of the vegetation cover connected with the increase in accumulated warmth in the Low Arctic. The authors rank them differently, and their positions vary greatly. The spatial shift of the studied boundary does not exhibit a specific direction, as indicated by the years of the publications, which may not accurately follow the ongoing climate change.

### 2.1 Geology and geomorphology

According to physical geographic zonation (Milkov, 1977), the studied area belongs to the Western Siberian region of the tundra zone, which includes the Yamal and Gydan provinces (Makunina, 1985; Rakovskaya & Davydova, 2001).

The terrain is formed by the West Siberian plate, covered by a thick layer of sediments. The Gydansky Peninsula is composed mainly of Cretaceous sediments, while the Yamal Peninsula is made up of Paleogene sediments (Markovskiy, 1975). The tundra of Western Siberia is situated in the continuous permafrost zone.

The area is predominantly a marine accumulative plain with a series of marine terraces descending to the Kara Sea. In general, it has a distinctively flat relief and is composed of gently sloping lowlands. The surface is weakly dissected by a network of mostly short and shallow valleys. Central and southern parts of the peninsulas are somewhat more elevated with maximum altitudes of 70–80 m asl on Yamal and 150–180 m asl on Gydansky. Cryogenic processes (frost cracking, solifluction, thermokarst, etc.) have caused the heterogeneity of the micro‐ and meso‐topography (various hummocks, polygons, circles, etc.), which, in turn, has caused the spatial complexity of the vegetation cover.

### 2.2 Climate

The West Siberian Arctic belongs to the subarctic and arctic climate zones (Alisov, 1956). Summers are short and cool; winters are long and cold with strong winds. The coldest month is January, with temperatures ranging from −21.1 to −25.8°C, while the warmest month is July, with temperatures ranging from +4.8 to +14.8°C (mean monthly temperatures were calculated for the study area using CHELSA v.2.1 (1981–2010), excluding extremes based on standard deviation; Karger et al., 2017, 2018). There is usually a low amount of annual precipitation (230–350 mm) and low evaporation. It is cloudy during all seasons. The share of direct solar radiation in the zone's heat balance is small. Heat mostly comes as diffuse radiation. Snow cover lasts from early October to mid‐June in open areas. The current climate warming rate in Western Siberia is 0.2–0.6°C/10 years (IPCC, 2021). The greatest change in seasonal temperatures is observed in spring and autumn. This leads to an increase in duration of the growing season (Kokorev & Sherstiukov, 2015; Tsaturov & Klepikov, 2012).

### 2.3 Soils

Soils in the West Siberian Arctic are generally moist acidic, gleyish, sandy, or loamy with thick peat horizon, which becomes thinner when moving into the High Arctic and disappears further north. Mires are very widespread occupying up to 30% of the area (Gvozdeckiy & Mikhailov, 1987).

### 2.4 Vegetation

The whole study area is located within the tundra vegetation zone and the tundra vegetation type is represented here in zonal habitats (Alexandrova, 1980; Gorodkov, 1935; Ilyina et al., 1985). Alexandrova (1980) distinguished two subtypes of the tundra vegetation type corresponding to the High and Low Arctic.

The most important circumpolar diagnostic feature of the High Arctic vegetation is an absence of *Betula nana* (dwarf birch), or, more generally, erect low shrubs. Hereafter, species names are given according to the Pan‐Arctic Species List, PASL (Raynolds et al., 2013). In the High Arctic of Western Siberia, the vegetation cover is dominated by the synusia of arctic and arctic–alpine prostrate dwarf shrubs (*Salix polaris*, *S. nummularia*, *Dryas octopetala*, and *D. punctata*). The High Arctic vegetation is represented by a variety of communities, such as forbs–sedge (*Carex bigelowii* subsp. *ensifolia*)–moss; forbs–*Dryas* spp.–moss; polar willow (*Salix polaris*)–cottongrass (*Eriophorum angustifolium*)–moss. An abundance of forbs is characteristic of these communities. The presence of *Eriophorum angustifolium* in zonal communities, and the absence of *Cassiope tetragona*, is a specific feature of the study region (Khitun & Rebristaya, 1998). In the south of the High Arctic subzone, vegetation cover is relatively closed, while further north it becomes discontinuous. However, non‐sorted circles are common already in the southern part.

Due to the harshness of climate conditions, the zonal and intrazonal habitats in the High Arctic are relatively similar. Vegetation types like shrub thickets and snowbed mesophytic meadows are absent. The most widespread intrazonal communities are homogeneous mires dominated by grasses (*Dupontia fisheri* and *Arctophila fulva*) or sedges (*Eriophorum russeolum* s.l. and *Carex concolor*) with mosses mostly of the Amblystegiaceae family. They occupy bottoms of shallow hollows, lake depressions, and river terraces. Polygonal tundra–mire complexes with *Carex concolor* dominating in wet hollows occur in river valleys. Colorful mesophytic meadows of arctic–alpine and arctic forbs on steep slopes with south or southwest aspect are also common (Rebristaya & Khitun, 1998).

The vegetation of the Low Arctic is characterized by the dominance of erect or hemiprostrate hypoarctic shrubs (30–50 cm high) and dwarf shrubs (*Betula nana*, *Salix glauca*, *Rhododendron tomentosum* subsp. *decumbens* (Labrador tea), *Empetrum nigrum* s.l., and *Vaccinium uliginosum* subsp. *microphyllum*). The height of shrubs decreases in the middle and northern parts of the Low Arctic, but their species diversity remains almost the same. A specific feature of the West Siberian Arctic is widespread Labrador tea‐dominated tundra along with dwarf birch tundra on watersheds and areas of tussock tundra with *Eriophorum vaginatum* on watersheds with poorer drainage (Gorodkov, 1935; Khitun, 1998; Telyatnikov et al., 2021a).

In intrazonal habitats, communities of shrubs, meadows, and mire vegetation types are found. Mire vegetation is the most widespread type and is represented both by homogeneous sedge (*Eriophorum angustifolium*, *E. russeolum* s.l., *Carex rotundata*, *C. chordorrhiza*, *C. rariflora*, and *Carex concolor*) – moss (Amblystegiaceae, Calliergonaceae, Scorpidiaceae, or Sphagnaceae)–mires and by polygonal tundra–mire complexes. The latter are a characteristic element of the Low Arctic landscape. High‐centered polygons are occupied by dwarf birch (alone or in combination with dwarf shrubs) or Labrador tea–cloudberry communities, invariable with a very thick moss turf (most often dominated by *Dicranum* spp. and *Polytrichum* spp.). Sedge–moss communities grow in surrounding wet hollows.

The presence of tall shrubs is also a diagnostic feature of the Low Arctic. Thickets of hypoarctic shrubs (*Salix lanata*, *S. glauca*) with hypoarctic forbs and grasses in the understory develop on the valleys' slopes and in wide shallow hollows (Khitun et al., 2015). They are 1.5–2.5 m high in the southern part of the Low Arctic, while usually no more than 1 m high (rarely up to 2.5) in the middle and northern parts (Dvornikov et al., 2015). Alder shrubs (*Duschekia fruticosa*) up to 3 m high can be found in riparian habitats in the southern part of the Low Arctic.

Meadow communities on steep sandy and loamy slopes or high sandy floodplains are the most colorful and diverse ones. They are usually dominated by arctic–alpine forbs (*Cerastium arvense*, *Polemonium boreale*, *Festuca rubra* subsp. *Richardsonii*, *Armeria scabra*, *Trisetum spicatum*, *Thymus reverdattoanus*, *Pachypleurum alpinum*, *Dianthus repens*, *Hedysarum hedysaroides* subsp. *Arcticum*, *Castilleja arctica*, and *Tanacetum bipinnatum*), which are not that widely represented anywhere else in the Low Arctic. Snowbed meadow communities with an abundance of mesophytic forbs (*Trollius asiaticus*, *Antennaria villifera*, *Astragalus alpinus*, *Pedicularis oederi*, *Sibbaldia procumbens*, *Omalotheca supina*, and *Carex lachenalii*) and prostrate dwarf shrubs (*Salix polaris*, *S. arctica*) develop at the feet of slopes in the Low Arctic. Wet meadows with dominating hypoarctic grasses and sedges (*Calamagrostis canadensis* subsp. *langsdorffii*, *C. neglecta*, *Poa pratensis* subsp. *alpigena*, *Carex concolor*, *Polemonium acutiflorum*, *Petasites frigidus*, and *Epilobium palustre*) can be found in the river floodplains.

### 2.5 Flora

The study area belongs to the Yamal–Gydan province of the Arctic floristic region (Figure 1; CAVM Team, 2003). There are about 450 vascular plant species in the regional flora, belonging to 52 families and 164 genera (Koroleva et al., 2011). Poaceae, Cyperaceae, Asteraceae, Caryophyllaceae, Salicaceae, and Ranunculaceae are the families with the largest number of species (Khitun, 1998; Rebristaya, 2013). In the regional flora, the arctic phytogeographic group of species predominates (45%); boreal and hypoarctic species are presented almost equally (26% and 29%, respectively; Khitun, 1998; Khitun et al., 2007; Sekretareva, 2004). Circumpolar species (54%–65%) have absolute dominance among the longitudinal geographical groups. Eurasian species are also quite common (23%–26%). Siberian species are more abundant on Gydansky (15%–18%), while European species are more common in the south of Yamal (9.6%), but they almost disappear in the High Arctic on Gydansky (Khitun, 1998; Khitun & Rebristaya, 1998; Rebristaya, 2013). There are about 250 lichen species in the West Siberian Arctic, with *Cladonia*, *Peltigera*, *Pertusaria*, and *Lecanora* genera being the most diverse (Andreev, 1984, 1994; Herbarium SVER, 2021; Magomedova et al., 2006). The list of mosses consists of approximately 276 species from 98 genera and 33 families (Czernyadjeva, 2001; Voronova & Dyachenko, 2018); the list of liverworts includes 121 species (Potemkin, 1993).

The lists of species specifically distributed in the High or Low Arctic of Western Siberia are presented in Section 3.1.

## 3 MATERIALS AND METHODS

### 3.1 Criteria for the High and Low Arctic demarcation and the possibility of their application

Unified circumpolar criteria, used for geobotanical subdivision of the Arctic, are based on the structure of vegetation cover and climate, while regional specifics are reflected in the floral composition of the communities. Our analysis revealed a discrepancy in the High–Low Arctic boundary position caused by the difficulty of accurately locating the criteria used, and not due to the differences in these criteria. The main agreed‐upon diagnostic features are the complete disappearance of dwarf birches in the High Arctic, a sharp decrease in the proportion of the boreal element of the flora, and a change in the vegetation structure. According to the established concept, the High–Low Arctic boundary coincides with the July isotherm of +6°С (e.g., Alexandrova, 1980; Myalo & Leonova, 2004). However, the authors did not consider the exact position and shape of the isotherms, and the effect of climate change. These criteria are difficult to map without high‐quality RS data and special software products.

Common criteria of the High–Low Arctic subdivision:

1. The distribution of key species capable of marking the High–Low Arctic boundary in Western Siberia. The analysis of the previously mentioned numerous literary sources and an extensive number of regional works (Rebristaya, 2013; Walker et al., 2019; etc.) enabled us to identify species growing strictly within one of the considered Arctic subzones:
  - High Arctic: *Draba subcapitata*, *D. oblongata*, *Carex minuscula* (=*Carex concolor* × *C. subspathaceae*, northern hybridogenic race of *Carex aquatilis*, not accepted in PASL, but recognized by Russian botanists and has very distinct distribution in the West Siberian Arctic; Rebristaya, 1991), *C. ursina*, *Stellaria longipes* (=*S. edwardsii*; Sekretareva, 2004), *Cerastium alpinum*, *Silene involucrata*, *Ranunculus sulphureus*, *Papaver lapponicum* subsp. *jugoricum*, *Potentilla hyparctica*, *Eritrichium villosum*, *Micranthes tenuis*, and *Saxifraga hyperborea*.
  - Low Arctic: *Rhododendron tomentosum* subsp. *decumbens*, *R. tomentosum* subsp. *tomentosum*, *Salix phylicifolia*, *Rubus chamaemorus*, *Empetrum nigrum* s.l., *Arctous alpina*, *Trollius asiaticus*, and *Veratrum lobelianum*.
  We additionally selected species that enter adjacent subzones, but have a distinctive range optimum in one of them:
  c High Arctic: *Salix polaris*, *S. nummularia*, northern ecotype of *Carex aquatilis*, accepted in Russian literature as *C. concolor* (Sekretareva, 2004), *Sarmenthypnum sarmentosum*, *Alopecurus borealis* (=*Alopecurus alpinus*; Sekretareva, 2004), *Draba alpina*, *D. glacialis*, *D. fladnizensis*, *Cardamine bellidifolia* subsp. *bellidifolia*, *Eriophorum angustifolium*, *E. scheuchzeri*, *Luzula kjellmaniana* (=*Luzula tundricola*; Sekretareva, 2004), *L. nivalis*, *Micranthes nelsoniana*, *M. hieraciifolia*, *Parrya nudicaulis*, *Saxifraga cernua*, *S. hirculus*, *Eritrichium villosum*, *Packera heterophylla* (=*Senecio resedifolius*; Sekretareva, 2004), and *Tephroseris atropurpurea*.
  d Low Arctic: *Salix glauca*, *S. lanata*, *S. pulchra*, *Dryas punctata*, *Саrех bigelowii* subsp. *ensifolia*, *Vaccinium uliginosum* subsp. *microphyllum*, and *V. vitis‐idaea* subsp. *minus*.
2. The northern limit of *Betula nana*‐dominated communities. All authors unambiguously indicate the absence of dwarf birch in the circumpolar High Arctic communities (Alexandrova, 1980; Gorodkov, 1935; Lavrenko & Sochava, 1954; Yurtsev, 1994, etc.). SDM with the data on the sites with *Betula nana* and on the corresponding environmental factors and spectral unmixing method give opportunities to use this criterion.
3. An absence of shrubs and tall dwarf shrubs is one of the most important diagnostic signs that indicate the circumpolar transition to the High Arctic (Alexandrova, 1980; Elvebakk, 1999). There are only prostrate dwarf shrubs (<15 cm high) in the High Arctic communities. A map of vegetation height could be used to derive this criterion (Bartsch et al., 2020).
4. Climatic variables. In all the aforementioned works on subdivision of the Arctic, the July isotherm of +6°C (average temperature of the near‐surface air) is considered to coincide with the High–Low Arctic boundary. There are two justifications for this criterion. First, the temperatures of the near‐surface air can be mapped using RS data, for example, MODIS LST products. Second, a map of temperatures can be created using reanalysis data.
  During the summer season, the surface temperature in the Arctic exceeds the air temperature data recorded by meteorological measurements by approximately 2°C (Comiso, 2003; Raynolds et al., 2008). Previous studies (Raynolds et al., 2008, 2019) have indicated that the surface temperature provides a more accurate representation of the low tundra vegetation environment compared to temperature data obtained from a height of 2 m above the ground. In this article, we utilize both parameters, surface temperature (MODIS LST) and air temperature at a 2 m height (reanalysis data). Considering the disparity in scale and the relative nature of the temperature data used, the surface temperature can be employed to validate the patterns of air temperature at the study scale.
5. Intrazonal communities. According to Alexandrova (1980) and other authors, snowbed and floodplain meadows disappear in the High Arctic. These can be interpreted using spectral reflection of vegetation in the optical satellite images (e.g., Hope et al., 1993; Laidler & Treitz, 2003; Stow et al., 2008) and Circumpolar Arctic Vegetation Map (Raynolds et al., 2019).
6. Vegetation productivity is considered to be a complementary criterion by some authors. For example, according to Bazilevich et al. (1997), the transition from the Low‐to‐High Arctic is marked where the total phytomass decreases to values below 30 t/ha, and net primary production decreases to 2.9 t/ha per year. However, at present, there are no reliable data to determine the exact location of this parameter.

**FIGURE 3:**
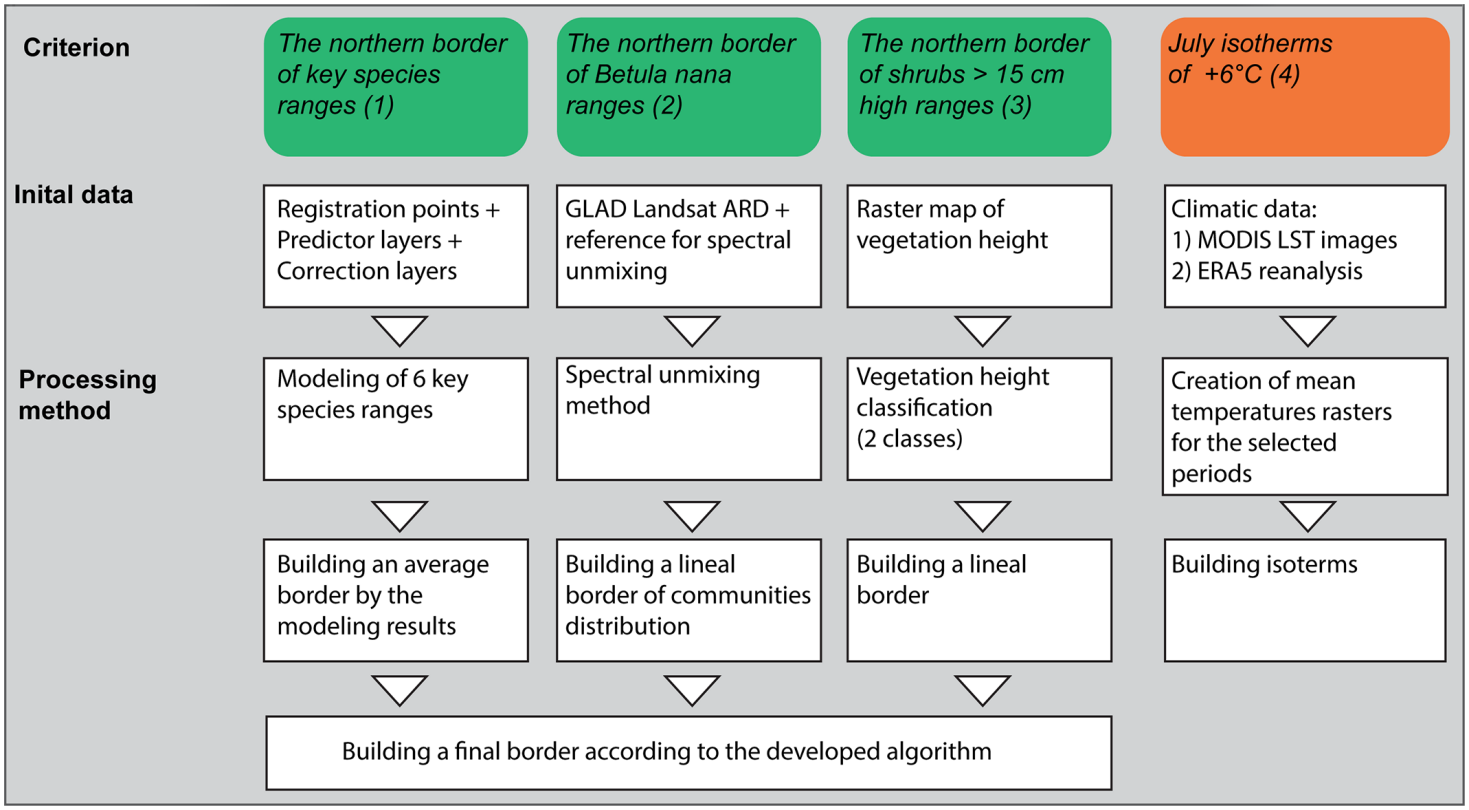
A conceptual workflow scheme of mapping the boundary between the High and Low Arctic. Green indicates criteria used for determining the position of the boundary, and orange indicates the criterion which was discarded.

By analyzing the subdivision schemes, existing RS data, and modern cartographical methods and programs, we defined the main criteria the authors used when dividing the subzones, and studied the possibilities of their implementation (Figure 3). Four criteria are possible to map using open data – (1) distribution of key species, (2) the northern boundary of the continuous distribution of *Betula nana*‐dominated communities, (3) the northern limit of distribution of shrubs and dwarf shrubs higher than 15 cm, and (4) the July isotherm. Due to its sparse distribution, the criterion based on the absence of intrazonal communities (5) could be effectively utilized for verifying of the final boundary position but not for mapping it. Despite the availability of GPP/NPP products based on remote sensing imagery, the vegetation productivity (complementary criterion 6) cannot be reliably mapped due to the absence of phytomass data required for the calibration of these products. Therefore, this criterion is not considered further.

### 3.2 Data on the vegetation structure and coordinates of the key species findings

**FIGURE 4:**
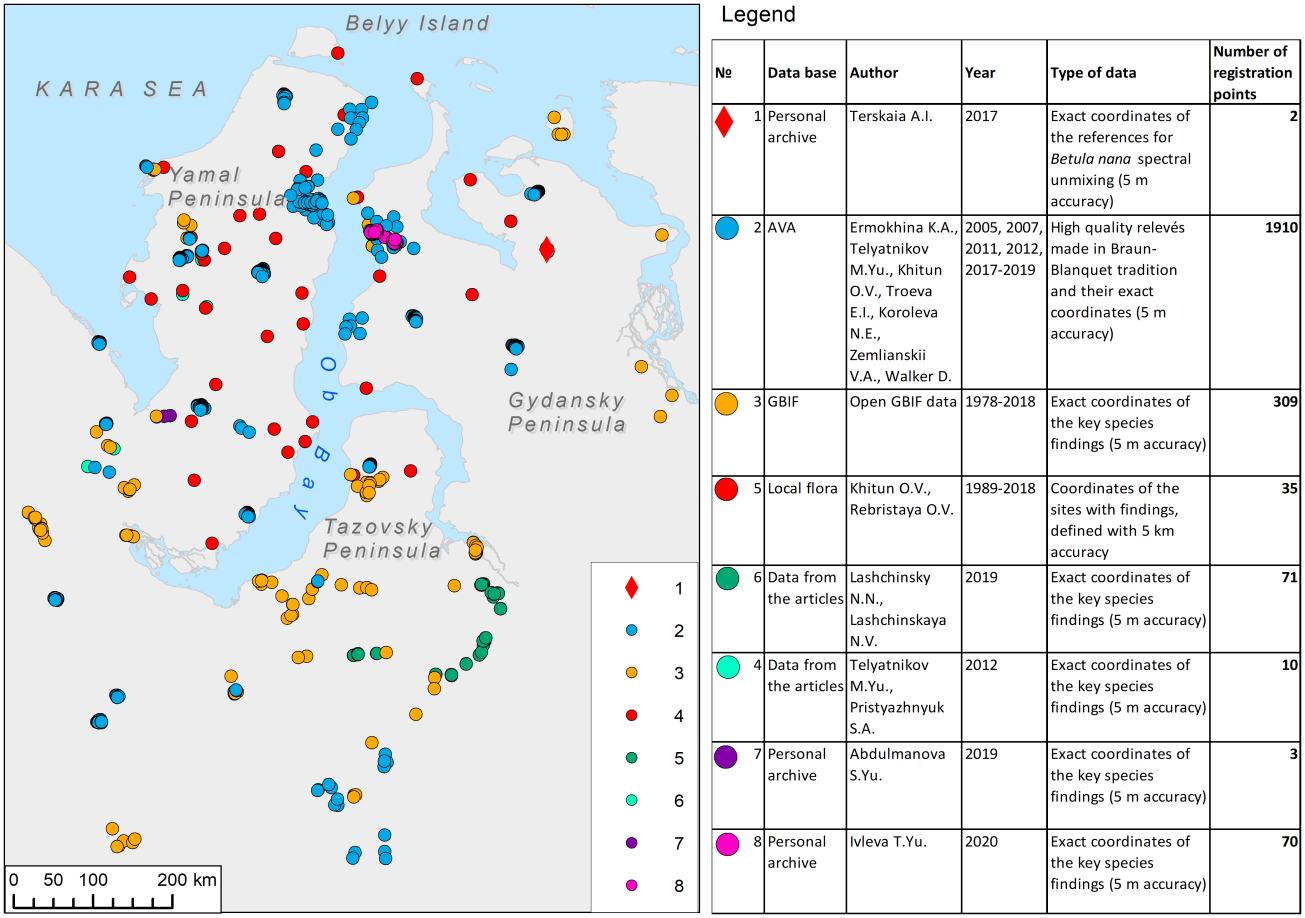
Structure and distribution of data on the findings of key species used for species distribution modeling

In our analysis, we used the information from the Arctic Vegetation Archive (AVA, https://avarus.space), Global Biodiversity Information Facility (GBIF, https://www.gbif.org), Herbarium of Lomonosov Moscow State University (https://plant.depo.msu.ru), published data on the local flora (Khitun, 2002, 2003, 2016; Khitun & Rebristaia, 2018; Khitun & Rebristaya, 1998; Rebristaya, 2013; Rebristaya et al., 1989; Rebristaya & Khitun, 1994), scientific articles (Lashchinskiy & Lashchinskaya, 2019; Telyatnikov & Pristyazhnyuk, 2012), and authors' personal archives (Figure 4).

The majority of data documenting species distribution were derived from geobotanical relevés made in accordance with the Braun–Blanquet tradition and standardized using the AVA protocol (relevés are available for download at the AVA website, https://avarus.space). Fieldworks were conducted during field seasons of 2005 (K. Ermokhina, 336 plots – Middle Yamal), 2007 (D. Walker, 10 plots – South Yamal, 25 plots – Middle Yamal), 2011 (K. Ermokhina, 128 plots – Middle Yamal), 2012 (K. Ermokhina, 9 plots – West Gydansky, 15 plots – North‐West Gydansky, 31 plots – North Yamal, 4 plots – South Yamal), 2017 (E. Troeva, 156 plots – North‐East Gydansky, 56 plots – North Yamal; M. Telyatnikov, 164 plots – North‐East Gydansky, 49 plots – North Yamal; O. Khitun, 229 plots – Middle Gydansky, 66 plots – Tazovsky; N. Koroleva, 73 plots – Middle Yamal, 217 plots – South Yamal; V. Zemlianskii, 81 plots – Middle Yamal, 87 plots – South Yamal), 2018 (S. Plusnin, 85 plots – footing of Yamal Peninsula; V. Zemlianskii, 80 plots – footing of Yamal Peninsula), and 2019 (K. Ermokhina, 9 plots – footing of Tazovsky Peninsula).

The relevés contain complete species lists (occasionally excluding liverworts), species' cover estimations, as well as vegetation and habitat characteristics. Typically, the described geobotanical plots (relevés) covered an area of 100 m^2^ and were distributed across a key site spanning 100 km^2^ to ensure comprehensive representation of different communities with a statistically significant number of plots. Approximately 3% of the relevés differed from this standard, either due to smaller plot sizes or distribution of plots in smaller key sites. To minimize the influence of the data structure (clustering of the points) on the analysis results, we thinned the selection by setting a minimum distance of 2 km between the findings. The selected relevés are rather evenly distributed across the investigated area and provide a representative sample of the vegetation diversity.

During fieldwork in 2017, we registered coordinates of homogeneous patches of about 900 m^2^ with a canopy density of 90%–100%, occupied exclusively by *Betula nana* (with no other shrub species). These patches became standards for interpreting Landsat images by the spectral unmixing method (Boardman, 1998).

### 3.3 Climate datasets

We used the bioclimatic CHELSA and EarthEnv datasets, and results of the analysis of MODIS images as predictor layers (raster data that demonstrate a distribution of some ecological feature) for SDM. CHELSA is an open‐access climate dataset of high spatial resolution for the Earth surface based on statistical downscaling of global circulation model (ERA) output with the Global Precipitation Climatology Centre displacement correction (Karger et al., 2017). A bioclimatic dataset, developed specifically for SDM, was used. These bioclimatic metrics are derived from the mean monthly and mean maximum temperatures and mean precipitation. They represent annual trends, seasonality, and extreme or limiting environmental factors.

The studied variables were supplemented with MODIS land surface temperature data (8‐day composites) for the month of July 2000–2019 with a spatial resolution of 1000 m (MOD11A2 MODIS/Terra Land Surface Temperature/Emissivity 8‐Day L3 Global 1 km SIN Grid V006 [Data set]). Mean temperatures of the land surface in January and July for the period 2000–2019 were calculated from the composites and were later used for SDM. We also used bioclimatic metrics from EarthEnv to complement the modeling set (Tuanmu & Jetz, 2015; www.earthenv.org).

The most complete set of layers includes 34 metrics (a detailed description of the full set is given in Appendix S2). To correct for latitudinal background selection bias resulting from the geographic coordinate system, we reprojected the spatial data to the Asia North Albers Equal Area Conic projection (ESRI:102025) and aggregated all raster layers to a spatial resolution of 2 × 2 km, which corresponds to the maximum coordinate uncertainty of the given species occurrences.

Spatial data on the environmental parameters are cross‐correlated, which can cause model instability and biased results (Dormann et al., 2013). MaxEnt (a program that was used further for SDM) is resistant to the influence of cross‐correlated predictors due to parameterization (Elith et al., 2011). We minimized multicollinearity between environmental variables by examining them for cross‐correlation and using only those below 0.75 correlation value threshold (Appendix S3a). We created three subsets of environmental factors: (1) automatically selected variables with Pearson correlation value less than 0.75 in the ArcGIS SDMToolBox program (Brown et al., 2017); (2) mutually orthogonal variables obtained by the principal component method in SAGA GIS software (Conrad et al., 2015), and (3) manually selected variables with Pearson correlation coefficient value <0.75.

### 3.4 Mapping the northern border of key species ranges (criterion 1)

Dwarf birch and a set of other species able to mark the High–Low Arctic boundary were chosen (*Salix lanata*, *Empetrum nigrum* s.l., *Rhododendron tomentosum* subsp. *decumbens* and *Rhododendron tomentosum* subsp. *tomentosum* (joint modeling), *Rubus chamaemorus*, *Vaccinium uliginosum* subsp. *microphyllum*, and *Arctous alpina*). An additional reason for choosing these particular species from those listed above was the presence of a sufficient number of registration points for modeling. To avoid spatially clustered occurrences, we applied a filtering process to the dataset and removed records that were closer to each other than 2 km. As a result, we utilized a total of 105 to 283 occurrences for each key species in our modeling analysis. The species that underwent examination but lacked an adequate number of known locations for realistic distribution modeling are listed in Section 3.1 (The distribution of key species).

We applied the presence background maximum entropy method for SDM implemented in the MaxEnt program as widely used in ecological and biogeographic studies (Phillips et al., 2004, 2017). To address spatial bias, a “bias‐file” was generated for correcting background selection (Phillips et al., 2009). This involved creating buffer zones with a radius of 2 km around all recorded points of selected key species. Only points within these buffer zones were used to generate a background sample consisting of 10,000 points. The MaxEnt modeling approach was employed with default options, including a regularization multiplier value of 1 and the use of linear, quadratic, and hinge features. For each of the chosen species, we constructed models with one full set and three reduced subsets (Appendix S3b). Results of the modeling are presented in Appendix S4.

The assessment of the models' quality was based on the AUC criterion (area under receiver operating characteristic curve; Araújo et al., 2005, Fielding & Bell, 1997). All the models obtained meet the formal quality requirements (AUC ranged from 7.36 to 9.2, Appendix S4) and could be used to define the northern boundary of the species ranges. We selected the SDM results with an expert set of layers for further analysis. Final models were limited by the threshold value of “Maximum test sensitivity plus specificity” calculated in MaxEnt (Liu et al., 2013).

To obtain an average boundary for all models, we calculated a standard deviation raster for all eight species' range rasters. We used normalized rasters for calculations, where the value of “0” was assigned to each cell with a value below the threshold. Minimum values of the standard deviation corresponded to the areas with no species occurrence probability, that is, to the areas where all eight species are below the distribution threshold. In order to define the border, the standard deviation with values below 10 was removed. The obtained line corresponds to the averaged northern boundary of the key species distributions.

### 3.5 Mapping of the northern border of continuous distribution of *Betula nana*‐dominated communities (criterion 2)

Areas with *Betula nana* occurring in plant communities were chosen according to Landsat image interpretation made with GLAD Landsat ARD product (Potapov et al., 2020). The latter was prepared by combining 16‐day composites with radiometric correction, normalized brightness, and cleared of clouds and haze. Interpretation was carried out in the ENVI software package using the spectral unmixing method (Meer, 1999). The method finds a reference spectrum in a pixel of a multispectral image based on the assumption that each pixel is a mixture of the known spectral signal of the sought object (reference) and random unknown background objects. Exact coordinates of homogeneous stands of *Betula nana* recorded during fieldwork in 2017 were used as references. Among spectral unmixing algorithms, we chose the method of mixture tuned matched filtering (MTMF; Boardman, 1998). This algorithm maximizes the signal of target spectra (reference) and suppresses the signal of unknown (unspecified) spectra. The algorithm resulted in two images: (1) the probability of the target presence; and (2) the probability of its absence. These images were then analyzed together and only pixels with high probability values of presence and low probability values of absence were selected. Thresholds were calculated automatically based on the probability distribution.

**FIGURE 5:**
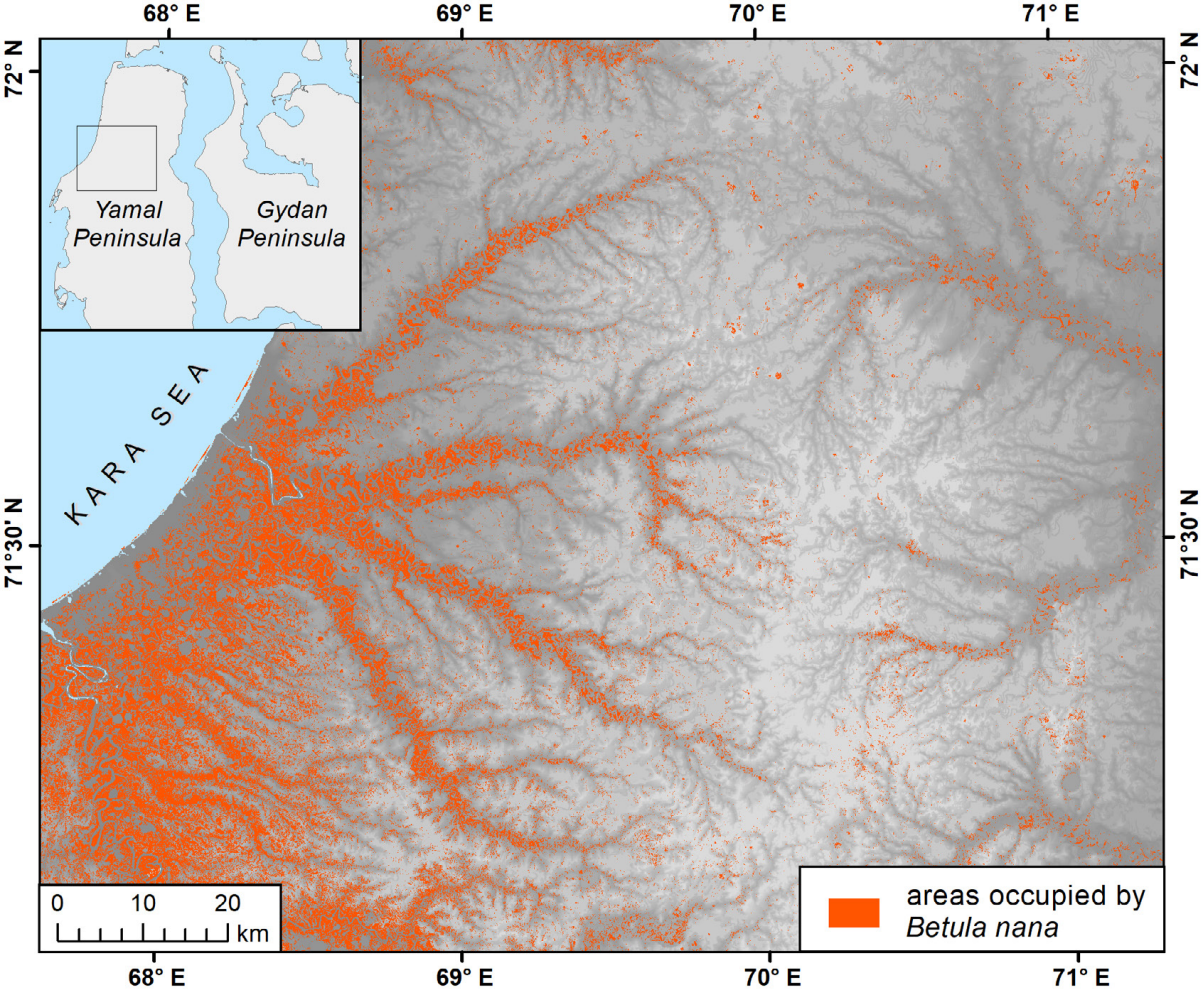
Distribution of *Betula nana*‐dominated communities according to the results of spectral unmixing (fragment of total area analyzed). The underlying map shows topography features (Digital Elevation Model provided by http://viewfinderpanoramas.org).

The accuracy of the spectral unmixing algorithm is generally up to 90% when using hyperspectral imagery (Villa et al., 2011). The accuracy of the results of *Betula nana* spectral unmixing was validated by comparing it with the AVA relevés; the analysis showed a good correspondence between them. The results of *Betula nana* detection by the spectral unmixing method are shown in Figure 5. The northern boundary of *Betula nana*‐dominated communities' continuous distribution at a scale of 1:1,000,000 was obtained as a continuous line of 1 × 1 km pixels occupied by *Betula nana* for more than 60%.

### 3.6 Mapping the northern limit of distribution of shrubs and dwarf shrubs more than 15 cm high (criterion 3)

This criterion was obtained from the vegetation height map (Bartsch et al., 2020). The initial spatial resolution of the map was 20 m. A transition to the scale of 1:1,000,000 was achieved by selecting pixels with ≥60% cover of shrubs and dwarf shrubs more than 15 cm high, using the previously created regular 1 × 1 km grid. The northern border of the continuous distribution of such pixels was automatically defined through pixel vectorization and was subsequently visually edited to address imperfections, such as broken and curly lines, and to obtain a continuous smooth line.

### 3.7 Assessment of the impact of human activities on the distribution of biotic delimitation criteria

In order to assess the influence of human activities on the distribution of the delimitation criteria, a cartographic comparative analysis was conducted in ArcGIS. This analysis involved comparing the criteria's boundaries with maps illustrating the anthropogenic transformation of ecosystems. Considering the study region, there were limited alternatives available. Correlation analysis was performed to examine the alignment of boundaries' contours with mapping units of the global record of annual terrestrial Human Footprint dataset from 2000 to 2018 (Mu et al., 2022), the Geographic distribution of global agricultural lands in the year 2000 (Ramankutty et al., 2008), and a map depicting sandy outcrops influenced by grazing reindeer published by Golovatin et al. (2012).

### 3.8 Generating July isotherms (criterion 4)

**FIGURE 6:**
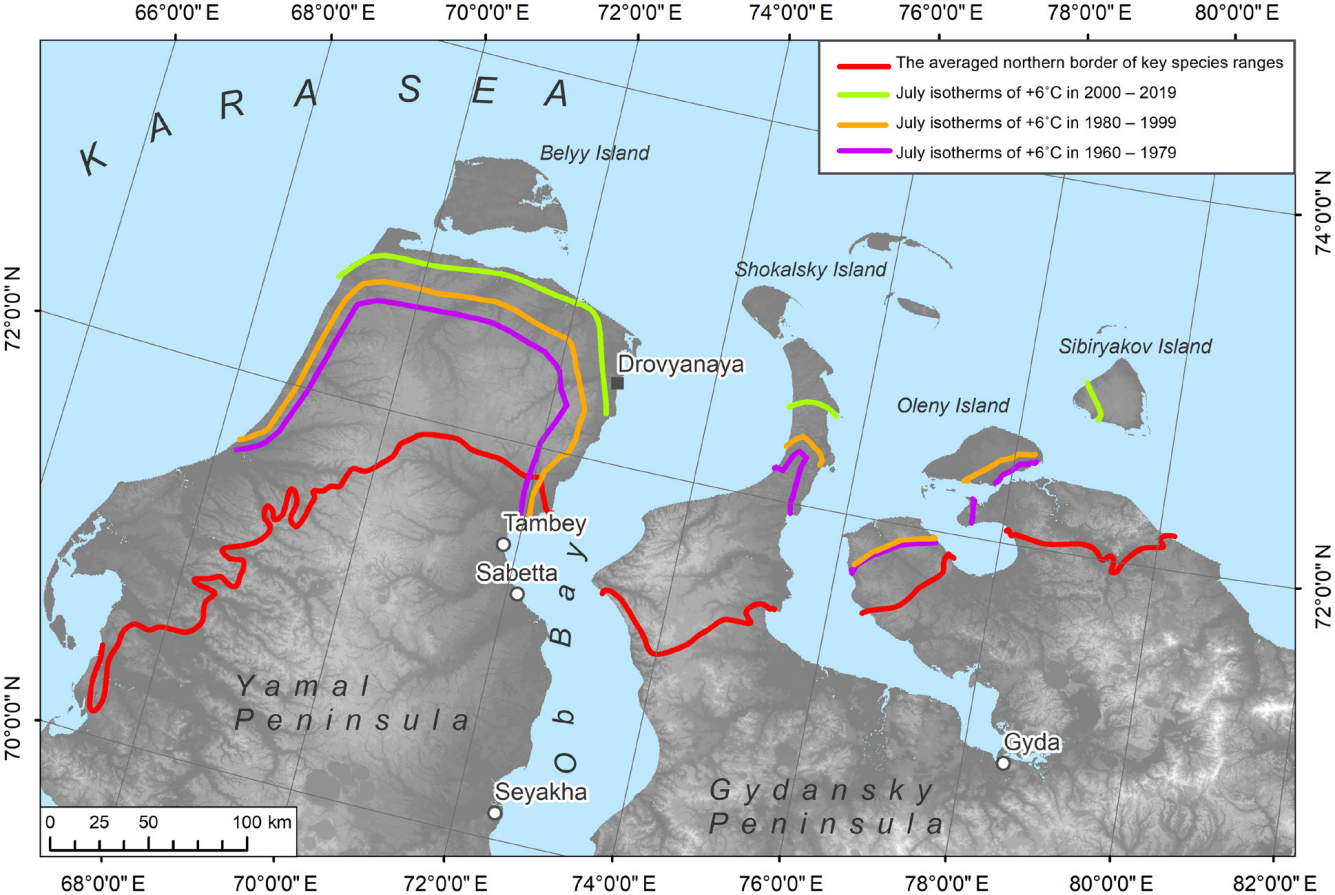
The averaged northern limit of key species ranges and the location of July isotherms of +6°C in 1950–1979, 1980–1999, and 2000–2019. The underlying map shows topography features (Digital Elevation Model provided by http://viewfinderpanoramas.org).

July isotherms were generated in ArcGIS according to the ERA5 mean monthly air temperature reanalysis data for three time periods: 1950–1979, 1980–1999, and 2000–2019 (https://cds.climate.copernicus.eu; Figure 6). These data have a spatial resolution of 0.1 × 0.1 degree, which corresponds to approximately 4 km in the north and around 10 km in the southern extent of the mapping area. We also employed data on land surface temperature distribution (MODIS) to visually verify the patterns of air temperature at the study scale.

The isotherms revealed that currently, the July isotherm of +6°C is approximately 100 km further north than the limit of key species distributions. Hence, this criterion was excluded when we generated the summarizing boundary between the High and Low Arctic.

### 3.9 Drawing the refined boundary between the High and Low Arctic

**FIGURE 7:**
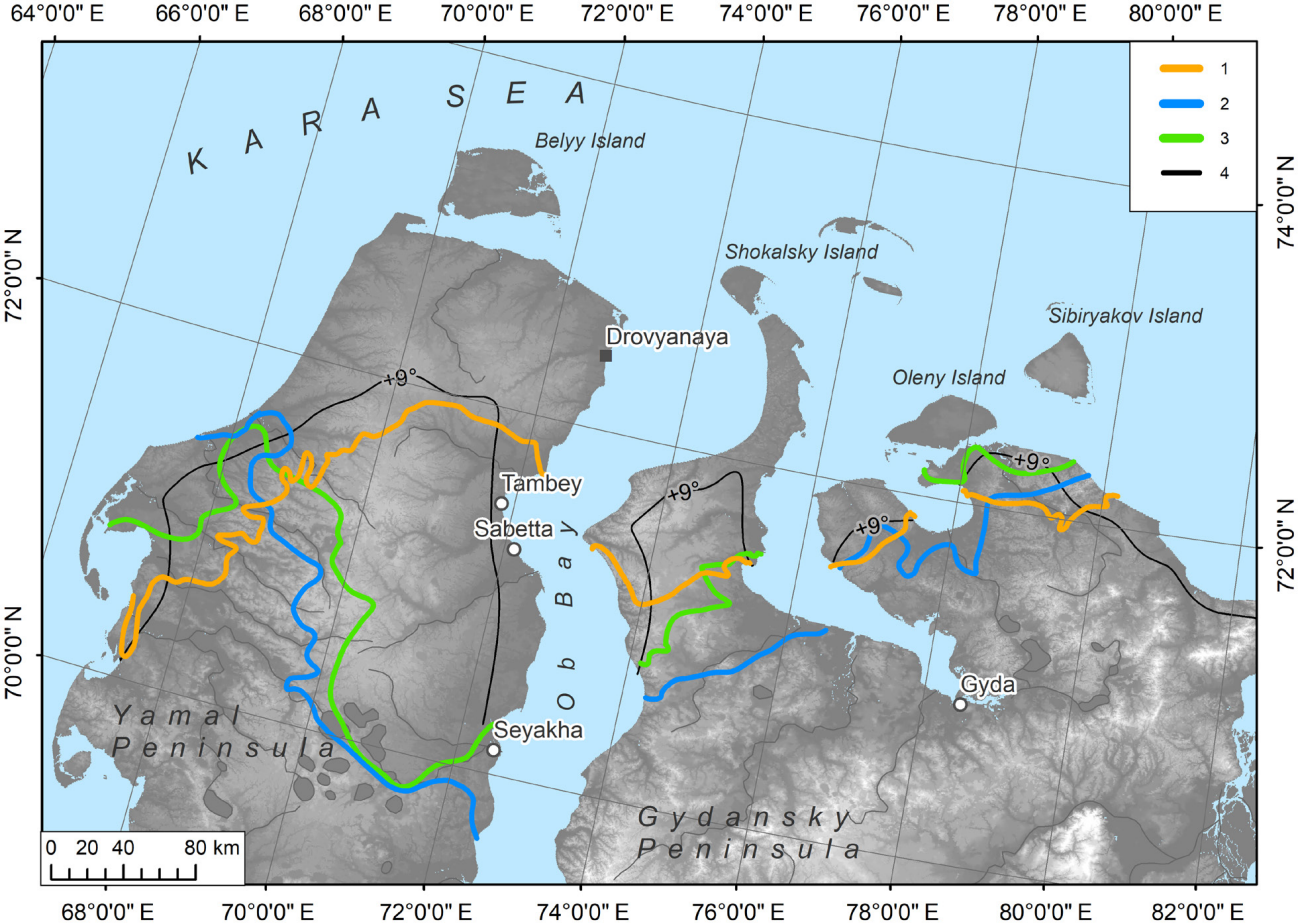
The position of (1) the averaged northern distribution limit of key species, (2) the northern limit of distribution of shrubs and dwarf shrubs higher than 15 cm, (3) the northern boundary of the continuous distribution of *Betula nana*‐dominated communities, and (4) the July isotherm +9°С (the climate variable that best matched other mapped boundaries; generated using ERA5, 2000–2019). The underlying map shows topography features (Digital Elevation Model provided by http://viewfinderpanoramas.org).

Figure 7 shows the borders which we obtained by mapping the above‐mentioned three biotic criteria used in our study. The redefined boundary between the High and Low Arctic integrates all the criteria borders obtained. The algorithm for its delineation is the following:

1. If the borders of all three criteria are located within a 25 km corridor, then the boundary is drawn in the middle of it;
2. If the northernmost criterion's border is located farther than 25 km corridor from the other two, or there is a distance of more than 25 km between all the lines, then we draw the final border along the northernmost one in order to meet all the criteria;
3. if one line is located more than 25 km south of the others, and the middle and northern lines are less than 25 km apart, then the final boundary line is drawn in a corridor between two northern lines.

The new boundary between the High and Low Arctic was visually checked for consistency with the Digital Elevation Model provided by http://viewfinderpanoramas.org. We used the raster version of the CAVM (Raynolds et al., 2019) for visual analysis of vegetation structure and localization of intrazonal communities (criterion 5). Verification confirmed the absence of significant controversial decisions during the implementation of the developed algorithm of automatic boundary localization and its application efficiency.

### 3.10 Analysis of biodiversity distribution in the boundary's neighborhood

To estimate species richness, we utilized 1089 geobotanical relevés with complete species lists. Liverworts were occasionally excluded from these lists, which, regrettably, is a common practice. However, this exclusion does not noticeably affect the values of total species richness. These relevés were collected by K. Ermokhina from 2005 to 2012, during the 2017 expedition, and along the EAT transect (Walker et al., 2019). They form part of the AVA data described in Section 3.2. For the biodiversity analysis, relevés located at the southernmost extent of the study region were excluded.

For each relevé, the richness of vascular plants, mosses, lichens, and total species richness (excluding liverworts) were calculated. The distance from the new High–Low Arctic boundary to the coordinates of each relevé was measured along the North–South direction. Subsequently, box plots were constructed to visually represent the biodiversity values based on the distance from the new boundary. All the analytical steps were performed using R (version 4.2.2; R Core Team, 2021); plots were made with ggplot2 package (Wickham, 2016).

## 4 RESULTS

There are four main common criteria researchers use to define the High–Low Arctic boundary – distribution of key species, the northern limit of distribution of shrubs and dwarf shrubs higher than 15 cm, the northern boundary of the continuous distribution of *Betula nana*‐dominated communities, and the July isotherm +6°C.

All the borders of key species ranges that were obtained by SDM are situated within a 30–50 km corridor (wider on the Gydansky Peninsula; Appendix S4). *Salix lanata* and *Rubus chamaemorus* ranges have expanded farther north than others. The northern distribution limit of *Betula nana*‐dominated communities (except the occurrence in intrazonal habitats within large river valleys) is close to the modeled northern border of dwarf shrubs higher than 15 cm and the averaged northern border of the key species ranges (Figure 7).

An exception is the eastern part of Yamal. Here, we question if human impact could significantly change distribution of the delimitation criteria. The actual position of dwarf birch‐dominated communities and northern limit of distribution of dwarf shrubs higher than 15 cm are 100–115 km farther south than the border obtained by SDM and verified by field relevés. Such a significant shift to the south could be associated with the transformation of vegetation due to the long‐term reindeer overgrazing (in Scandinavia: Olofsson et al., 2009) and expansion of the gas industry infrastructure that causes degradation of shrub vegetation in this part of the peninsula (Kryazhimsky et al., 2011; Skipin et al., 2014). Another important factor affecting the vegetation cover in the eastern part of Yamal is the sandy sediments that are widespread here and prone to deflation. The deflation can start due to both natural and anthropogenic factors and dramatically transform local landscapes over very large areas, inhibiting shrubs and dwarf shrubs development (Ermokhina & Myalo, 2012; Magomedova et al., 2006).

**FIGURE 8:**
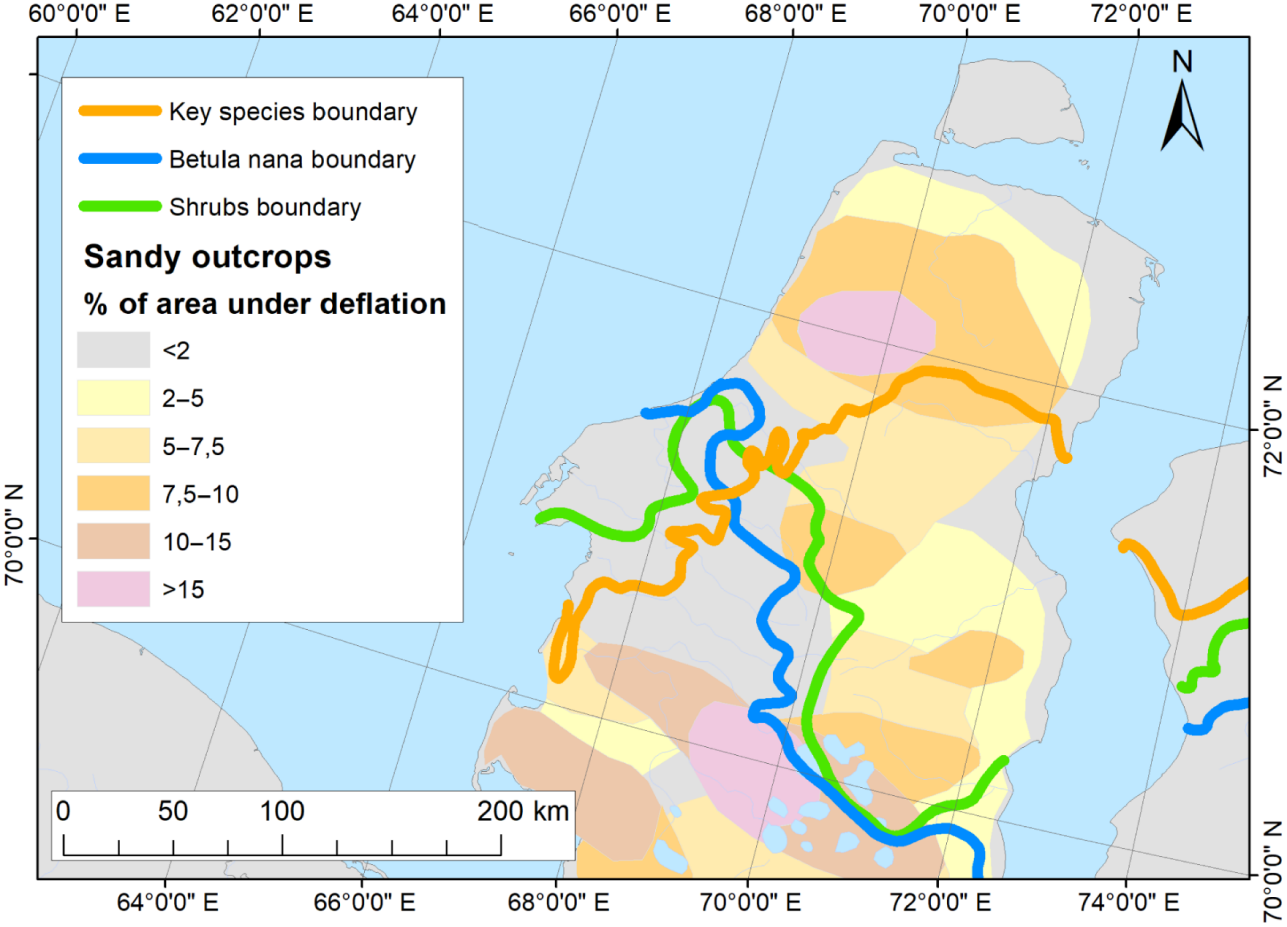
Impact of reindeer grazing on vegetation structure at the eastern part of Yamal. The averaged northern distribution limit of key species (orange line), northern boundary of the continuous distribution of *Betula nana*‐dominated communities (blue line), and the northern limit of distribution of erect shrubs higher than 15 cm (green line) are shown on the map of sandy outcrops formed under the influence of grazing reindeer, published by Golovatin et al., 2012.

In our opinion, the spatial gap, observed in the characteristics of the flora and vegetation of the studied area, is mainly explained by anthropogenic influence, specifically pastoral activities (Figure 8). The high number of reindeer in herds (up to 10,000 individuals) and the absence of pasture rotation result in a significant transformation of vegetation. This effect may be further amplified by the prevalence of deflating sandy soils. Upon analyzing the boundaries of the criteria, we found no correlation between the mapping units of the global record of annual terrestrial Human Footprint dataset from 2000 to 2018 (Mu et al., 2022) and the Geographic distribution of global agricultural lands in the year 2000 (Ramankutty et al., 2008). The maps depicting human footprint clearly illustrate industrial activities in the West Siberian Arctic, but their impact appears to be local and unlikely to cause significant changes in the flora and vegetation at the scale of our study. The map of agricultural lands lacks comprehensive information for the entire Arctic and is therefore not applicable for analyzing the study region. In order to meet all considered criteria, we relied on the results of SDM and field data when drawing the High–Low Arctic boundary in the east of Yamal (the algorithm is described in Section 3.9).

Climate usually changes significantly faster than the reaction of tundra vegetation, hence, ecosystems require a longer period to transform (Matveeva, 1998). However, one of our questions was if the widely accepted temperature criterion, based on the July +6°С isotherm, is still relevant given the ongoing climate change. We got an unexpected answer.

**FIGURE 9:**
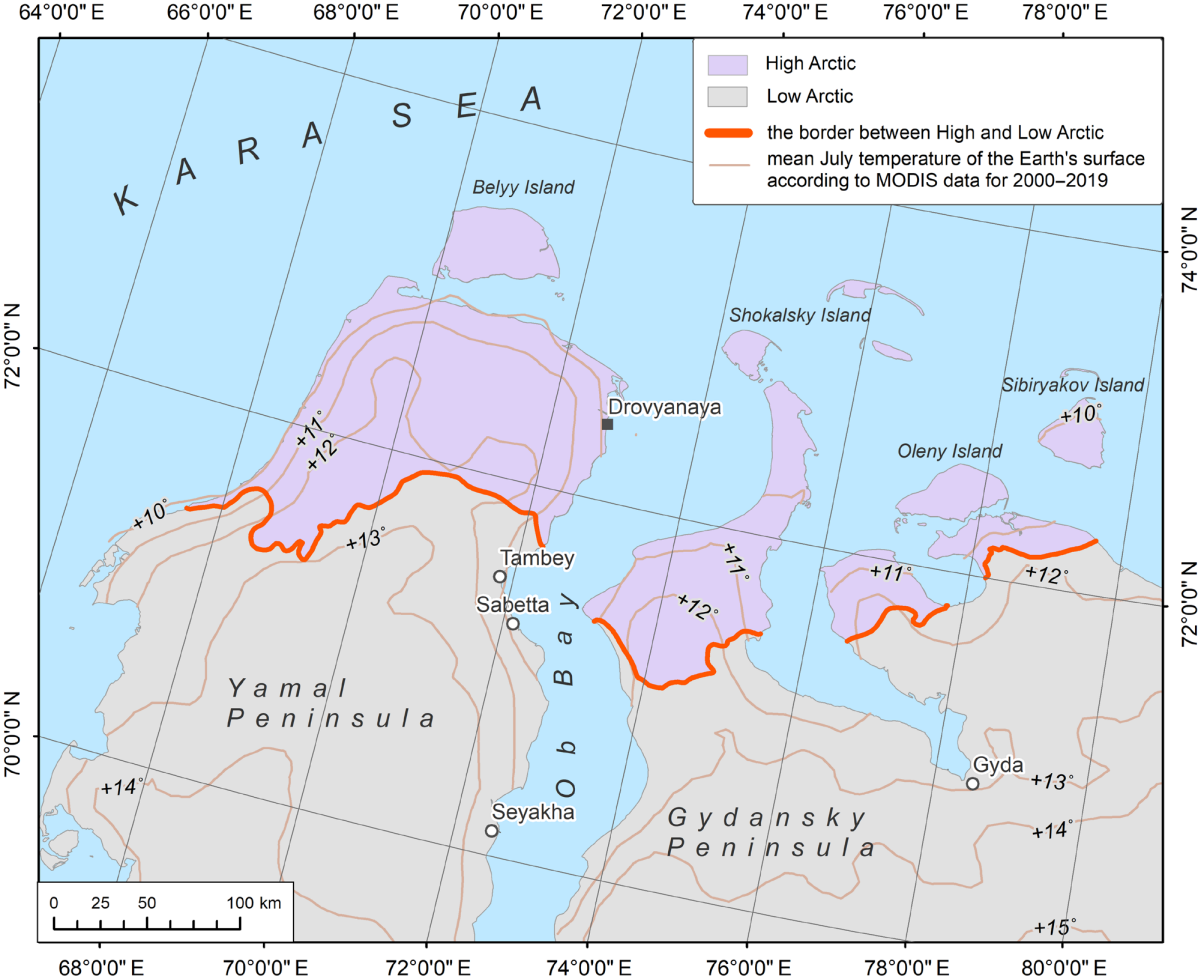
The refined boundary between the High and Low Arctic in Western Siberia and mean July temperatures of the land surface (MODIS, 2000–2019).

Our analysis of the climate data shows that there is no direct correlation between the average July isotherm +6°С (estimated for air at 2 m height) and the High–Low Arctic boundary in Western Siberia (Figure 6). In fact, it is the July +9°C isotherm that more closely coincides with the newly established boundary position (Figure 7). The isotherm of +6°С based on the average temperature values of the last 20 years (2000–2019) according to the ERA5 is located 160–200 km further north than is shown on small‐scale maps in regional atlases (e.g., Kasimov et al., 2018; Tryoshnikov et al., 1985). Furthermore, the position of the boundary between the High and Low Arctic does not coincide with the averaged position of the July +6°С isotherm for the period from 1960 to 1999 (Figure 6). This probably indicates that this temperature parameter has never correlated well with this geobotanical boundary in Western Siberia. According to the MODIS data averaged over the last 20 years (Figure 9), the July +12°С isotherm for the land surface correlates with the newly obtained position of the High–Low Arctic boundary quite well. The analysis indicates that the difference between July air and surface temperatures in the study region, for the period 2000–2019, is about 3.5°C. This exceeds the estimated values for Alaska from 1981 to 2000 (Raynolds et al., 2008) by approximately 1.5°C.

Based on our research, we do not recommend using previously accepted isotherms as direct indicators for locating the High–Low Arctic boundary. Our approach for defining a position of the High–Low Arctic boundary follows the traditional geobotanical criteria but includes a comprehensive analysis of modern open data on vegetation cover (results of the interpretation Landsat images, vegetation, and vegetation height maps), on the ecological relationships between vegetation and environmental factors (results of the ecological ordination of the described syntaxa; Telyatnikov et al., 2019a, 2019b, 2021a, 2021b), and species composition of plant communities (results of SDM and standardized relevés of the AVA). Data on topography (Digital Elevation Model provided by http://viewfinderpanoramas.org) and climate (CHELSA, MODIS) were also considered through modeling and verification. The result position of the High–Low Arctic boundary is shown in Figure 9.

Application of the developed approach clarified the boundary's position in the study region and revealed that it is located further north than it had previously been defined. The boundary we established is located at latitude of 71°21′ N on the west coast of Yamal, from there it gradually rises to 71°55′ N in the central part of the peninsula, and then descends to 71°40′ N on the east coast. On the west coast of the Gydansky Peninsula, the border lies at 71°45′ N, then it gradually shifts southwards to 71°20′ N. To the east, it lies at 71°50′ N and reaches 72°18′ N on the east coast of Gydansky. Thus, the boundary between the High and Low Arctic on the Gydansky Peninsula is located farther north than on the Yamal Peninsula and shifts gradually northwards in direction of the Taimyr. This tendency was also noted in earlier maps (Figure 2).

The comparison between the positions of the High–Low Arctic boundary in the literature and from cartographic sources (Figure 2) and the boundary we delineated shows that the latter lies closest to the position suggested by Khitun et al. (2007), however, that line was drawn 50–90 km south on Yamal and 5–50 km further south on Gydansky. By its shape on Yamal, our boundary most closely resembles the boundary of subzone C drawn by CAVM Team (2003), but it is 70 km farther north. The position of the boundary on Gydansky obtained by us deviates markedly from all previously suggested: 100 km farther north than on the CAVM, and 50–100 km farther north compared to other sources. Field observations (Khitun, 2003; Telyatnikov et al., 2019b) confirm that typical northern Low Arctic communities occur much further north than would be expected according to accepted delimitation schemes (Alexandrova, 1980; CAVM Team, 2003; Yurtsev, 1994).

**FIGURE 10:**
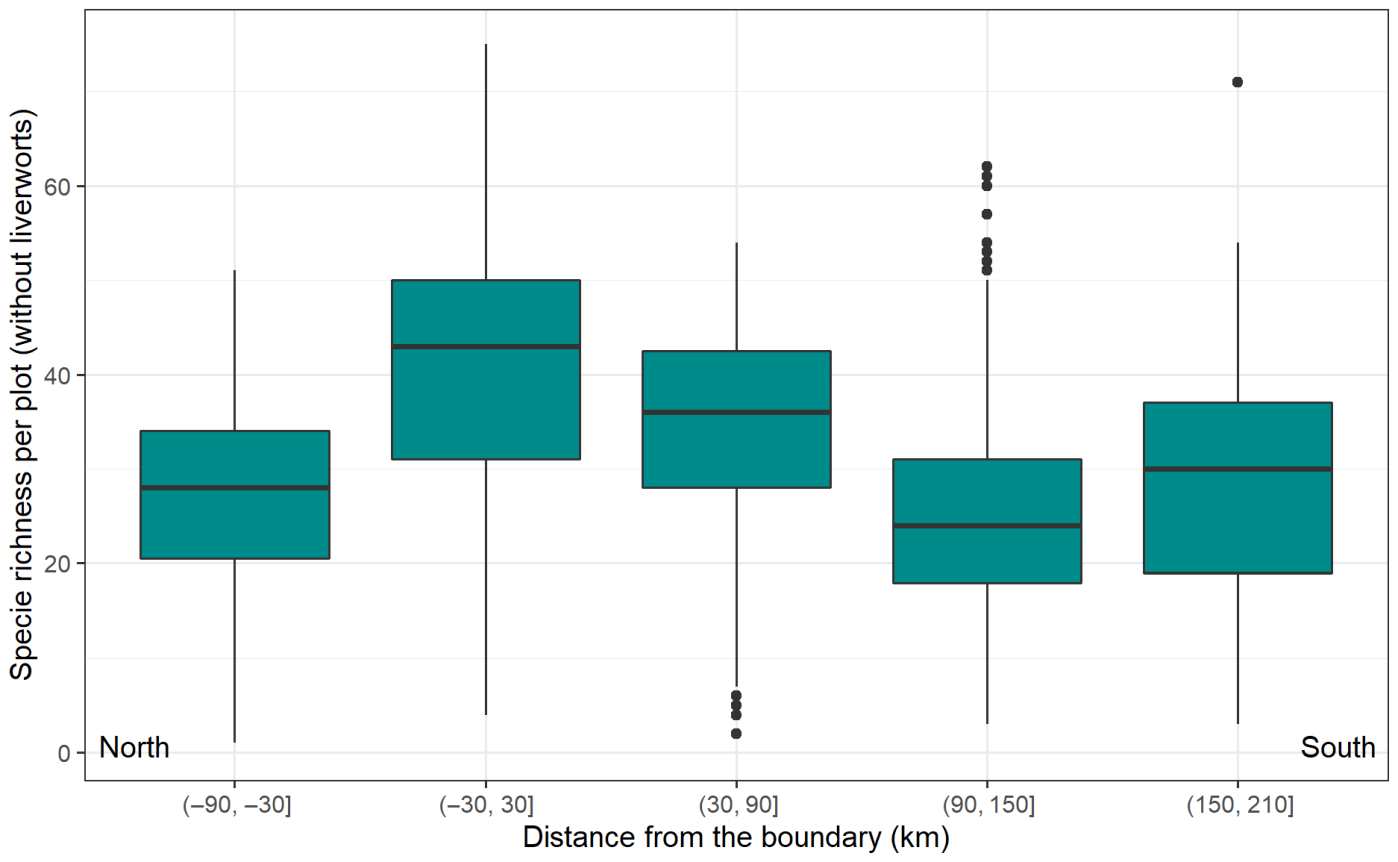
Total species richness (vascular, mosses, and lichens, without liverworts) depending on the distance from the High–Low Arctic boundary. Numbers along the x‐axis indicate the range of distances from the boundary for plots within each box, with negative numbers indicating sites north of the boundary and positive number sites south of the boundary. Plots shown in the (−30, 30) box plot are all within 30 km of the boundary and have the highest species richness.

Our location of the boundary clearly captures biodiversity peak in transition between the High and Low Arctic (Figure 10; Appendix S5). Biodiversity of vegetation increases at the boundary as communities contain species with southern and northern range optima. The peak is most clearly expressed in the diversity of vascular plants. The diversity of mosses and lichens increases more gradually while approaching the boundary. The analysis of species distribution shows that the width of the boundary on the ground is about 60 km. Appendix S5 presents the results of the biodiversity analysis in detail.

## 5 DISCUSSION

We propose the utilization of three biotic criteria related to species distribution and structural characteristics of vegetation for the division of the High and Low Arctic. The concordance and reliability of the floristic and vegetation criteria were demonstrated (Figure 7). We suggest excluding the temperature criterion, which was previously accepted in vegetation science, due to its estimated invalidity in at least one of the Arctic floristic provinces (Figure 6).

Our research indicates that local industrial impact does not alter the distribution of the criteria at the study scale. On the other hand, activities that transform extensive areas, such as reindeer grazing, may affect the distribution of criteria based on vegetation characteristics, while having a minimal impact on the distribution of key species (Figure 8). In such cases, the developed algorithm for delineating the High–Low Arctic boundary (described in Section 3.9) effectively removes any bias.

The new information on isotherms that coincide with the High–Low Arctic boundary (Figures 7 and 9) has great importance since temperature is the main factor that shapes arctic vegetation (e.g., CAVM Team, 2003; Epstein et al., 2017). This is also supported by our species distribution models: the number of days with temperatures above +10°C and the average annual air temperature have the most impact on the key species distribution. However, we do not recommend using the exact isotherms estimated in this study as direct indicators for locating the High–Low Arctic boundary in other Arctic regions without prior testing.

The proposed analytical approach represents a significant improvement over previous contemplative methods. By analyzing remote sensing data, detailed information on vegetation cover can be obtained for the entire Arctic. Species distribution modeling allows for the integration of multiple environmental factors and species‐specific responses. The use of SDM and RS data analysis enhances the accuracy of the mapping process.

Another strength of this approach is its departure from the temperature criterion previously used to define the High–Low Arctic boundary. By incorporating biotic criteria, the approach captures a more comprehensive understanding of the transition. Vegetation and flora directly respond to environmental conditions and provide valuable insights into the ecological changes occurring in the Arctic.

The new boundary explicitly highlights the peak biodiversity in the transition between the High and Low Arctic in Western Siberia (Figure 10, Appendix S5). The biodiversity distribution pattern indicates that the width of the boundary on the ground is approximately 60 km. The correlation could be similar in the other plain Arctic regions but would be expected to be sharper in mountain areas as bioclimatic parameters there change faster in smaller distances. This estimated regularity could help in locating biodiversity hotspots across the Arctic.

To minimize mapping bias, the selection of specific methods incorporated into the developed approach was based on their 1 km spatial resolution confidence, and the differences in data types and analytical mechanisms utilized. However, limitations of the developed approach exist. One such obvious limitation is the reliance on available data for mapping certain criteria. While the study demonstrates the feasibility of mapping vegetation metrics in Western Siberia using existing data sources, other regions may not be reliably analyzed due to the lack of data. We anticipate these problems for the Russian Far East. Additionally, quality of remote sensing and vegetation data introduces the potential for bias and errors.

The delimitation criteria based on the vegetation characteristics remain consistent throughout the Arctic, while the floristic criterion may vary. In a circumpolar context, the northern limit of *Betula nana*'s range can be relied upon as an indicator. However, in other floristic provinces, the distribution of different species may be associated with the boundary between the High and Low Arctic. For example, when assessing North America, the distribution of another dwarf birch species, *Betula glandulosa*, should also be taken into consideration.

Despite these limitations, the proposed approach represents a valuable improvement that provides a systematic and replicable framework for delineating geobotanical boundaries. The developed approach makes it possible to accurately map the High–Low Arctic boundary for the entire Northern Hemisphere using modern open vegetation, remote sensing, and climatic data. This standardized approach could enhance comparability across studies. Among the advantages of clearly defined and unified criteria for locating the High–Low Arctic boundary is the opportunity to apply and use it in climate and ecological modeling, as well as in landcover and biodiversity analyses and mapping. Future research should focus on regionally refining the approach, expanding data collection efforts, and investigating the monitoring implications of geobotanical boundaries mapping in the face of climate change.

## AUTHOR CONTRIBUTIONS

**Ksenia A. Ermokhina:** Conceptualization (lead); data curation (lead); formal analysis (equal); investigation (lead); methodology (equal); project administration (lead); writing – original draft (lead). **Anna I. Terskaia:** Data curation (equal); formal analysis (equal); investigation (equal); methodology (lead); writing – original draft (equal). **Tatiana Yu. Ivleva**: Data curation (equal); formal analysis (lead); investigation (equal); methodology (supporting); visualization (lead); writing – original draft (equal). **Sergey V. Dudov:** Data curation (equal); formal analysis (supporting); investigation (equal); methodology (equal); writing – original draft (equal). **Vitalii А. Zemlianskii:** Data curation (equal); formal analysis (equal); funding acquisition (lead); visualization (supporting); writing – original draft (equal). **Michael Yu. Telyatnikov:** Data curation (equal); investigation (supporting); validation (equal); writing – original draft (equal). **Olga V. Khitun:** Conceptualization (supporting); data curation (equal); investigation (supporting); validation (equal); writing – original draft (equal). **Elena I. Troeva:** Data curation (equal); investigation (supporting); validation (equal); writing – original draft (equal). **Natalia E. Koroleva:** Data curation (equal); validation (equal); writing – original draft (equal). **Svetlana Yu. Abdulmanova:** Data curation (equal); validation (supporting); writing – original draft (supporting).

## FUNDING INFORMATION

This work is supported by a Swiss Government Excellence Scholarship (2019.0075).

## CONFLICT OF INTEREST STATEMENT

The authors have no conflict of interest to declare.

## Supporting information


Appendix S1

Appendix S2

Appendix S3

Appendix S4

Appendix S5

## Data Availability

The data that support the findings of this study (including the GIS project and the raw dataset) will be openly available in the AVA at https://avarus.space upon the publication of the article. A copy of the open‐access part of AVA‐RU data is stored regularly on the external data archive Dryad, using a versioning system (Zemlianskii et al., 2023; https://doi.org/10.5061/dryad.5tb2rbp8d).
