# Supplementary material for: The High–Low Arctic boundary: How is it determined and where is it located?
Source: Ecol Evol. 2023 Sep 28;13(10):e10545. doi: 10.1002/ece3.10545 (PMC10539046; doi:10.1002/ece3.10545)
Supplement: Supplementary file 1 — Appendix S1 [file ECE3-13-e10545-s002.pdf]

Appendix 1. Glossary of geobotanical terminology used in the article, and Brief overview of research conducted in the West Siberian Arctic prior to the present study.

Glossary of geobotanical terminology

Biodiversity – the variety and variability of life on Earth. Biodiversity is a measure of variation at the genetic (genetic variability), species (species diversity), and ecosystem (ecosystem diversity) levels. At the *plant community* level (described by *relevés*), the **species richness** of certain groups (e.g., the number of vascular plant or lichen species) is distinguished as components of overall species diversity.

Ecological groups of terrestrial plants based on water requirements – **hygrophytes** (plants that grow in wet conditions), **mesophytes** (plants that live on an average supply of water), and **xerophytes** (plants with adaptations to survive in an environment with a lack of liquid water).

Flora – most general definition: a list of the plants of an area or period; more specific (as used in this paper) definition: historically formed complete set of species (taxa) of a certain territory (Yurtsev and Kamelin, 1991). Although it is not specified in definition, the term is normally applied to vascular plants. If referring to other groups, they are specified, for example, flora of bryophytes or flora of lichens. There is a hierarchy of floras on a geographical scale: planetary (flora of the Earth), continental (Flora of Eurasia, Australia, etc.), regional, local. **Local flora** – a special term and concept introduced by Russian botanists, meaning a minimal natural floral unit, a flora of a representative area (in the Arctic it is 100-300 km^2) where all habitat types are searched very thoroughly, and a complete list of species is revealed. The method of local floras was introduced for studying vast wilderness areas in northern Russia. For more details about the local flora approach, see Khitun et al., 2016; Yurtsev, Kamelin, 1991.

Geographical groups of plants (*syn*. **phytogeographical groups**) – groups of plants according to their geographical distributions. Analysis of the geographical structure of a flora means analysis of the compound and proportion of phytogeographical groups. In Russian Arctic geobotany traditionally latitudinal (or zonal) and longitudinal (or sectoral) distributions of species are considered separately. **Latitudinal geographical groups** of plants are as follows: **arctic** (it includes 3 subgroups: **proper arctic** species, growing only within the tundra zone; **metaarctic** species**,** growing in the tundra zone and in adjacent *subarctic* mountains; **arctic-alpine**, growing in the tundra zone and in mountain tundra elsewhere); **hypoarctic** (it includes **proper hypoarctic** species, with ranges within northern taiga and in Low Arctic; **hypoarctic-montane** species occurring in the *Hypoarctic* and also in the forest belt and mountain tundra in southern mountains); **borea**l (it includes **proper boreal** species with ranges within the taiga zone and **arctic-boreal** species with broad distribution in taiga and tundra). **Longitudinal geographical groups** of plants can be distinguished at different scales of detail, they reflect distribution within the longitudinal sectors of the continents. The most general are: **circumpolar** species, growing in the entire Arctic region; **Eurasian**, with broad ranges from Fennoscandia to Chukotka; **American,** distributed throughout the northern part of North America. Many more specific groups can be distinguished, such as west Eurasian, Asian, Middle Siberian, Amphi-Atlantic, Chukotkan-American, etc. Their names reflect distribution ranges. (More details in: Khitun et al., 2016; Koroleva et al., 2011; Tolmatchev, 1974).

Gross primary production (GPP) – the total amount of energy fixed by all the autotrophs in an ecosystem; the sum of net primary productivity plus respiration by autotrophs (GPP = NPP + R). It represents the fixed energy per unit area by the photosynthetic activity of plants before respiration; the total energy flow at the secondary level is not gross production but rather assimilation, because consumers use material already produced with respiratory losses.

Hypoarctic – territory from the northern taiga to the north of Low Arctic. In Russian terminology, it includes the northern taiga, southern tundra (*syn.* southern hypoarctic) and typical tundra (*syn.* northern hypoarctic) subzones (Yurtsev, 1966, 1994).

Intrazonal habitats – those not included in the group of zonal (see below), they exist due to local variations of topography, snow accumulation, soil diversity, and have microclimate that deviates from the general macroclimate associated with flat areas. Different slopes, depressions, floodplains, snowbeds are examples of intrazonal habitats. The intrazonal habitats form a mosaic of communities. Intrazonal communities are often relatively poor, but together they are responsible for about 80% of the total *species diversity* in the *local flora* or regional flora (Matveeva, 1998; Callaghan et al., 2004).

Intrazonal vegetation – vegetation, occurring in *intrazonal habitats*, it is a *vegetation type* that is included in the *zonal vegetation* but does not form an independent zone**.** It is divided into **proper intrazonal** (present only within certain zone), **extrazonal** (communities which are outside their distribution in zonal habitats on the adjacent territories) and **azonal** vegetation which is not connected with any particular zone (such as *snowbed meadows* and *mires*). In this paper we use the term “intrazonal” in the meaning that it is different from *zonal vegetation* (Matveeva, 1998).

Isotherm – a line on a map or chart of the Earth's surface or the air at a certain height connecting points having the same temperature. Isotherms are commonly used in geobotany to characterize ecological requirements of species and *plant communities*.

Mires – peat-forming wetlands that develop in areas with high rainfall or poor drainage, where the accumulation of dead plant material exceeds the rate of decomposition. Mires, in contrast to bogs, have groundwater feed.

Net primary productivity (NPP) – Gross primary productivity (the total amount of energy that plants produce) minus the energy plants use during cellular respiration (NPP=GPP-R).

Plant community or phytocoenosis – an elementary unit of vegetation, a totality of vascular plants, bryophytes, and lichens on a homogeneous part of the Earth’s surface, characterized by a certain composition and structure, which formed in the process of selection of species able to grow together in certain environments.

Plant functional types – sets of species with similar responses to the environment and with similar effects on ecosystem functioning. They could be more general (woody, non-woody, cryptogams) or more specified as trees, shrubs (perennial woody plants 0,8-6 m high, with several stems, in the Raunkier system belong to phanerophytes), dwarf shrubs (low 2-60 cm high, perennial plants with woody stems, usually without a prominent main stem, can have cushion-like form, in the Raunkier life form they belong to hamephytes), graminoids (grasses

and sedges), forbs (graminoids and forbs belong to hemicryptophytes and therophytes in the Raunkier system), bryophytes, lichens.

Phytomass – the total mass of living plant material in a given area.

Relevé – phytosociological sampling plot following the Braun-Blanquet method, synonymous Russian literature is "geobotanical description". At a sampling plot of a certain size (most common sizes in tundra are 1x1, 5x5, or 10x10 m), all present species of vascular plants, bryophytes, and lichens are recorded with their cover-abundance values. Additionally, the cover of different *plant functional types* and the height of vertical plant strata, along with basic abiotic data (topography and microtopography, aspect, inclination, soil), are recorded.

Snowbed meadows – herbaceous *vegetation* growing in the area of snowfields confined to landform elements protected from wind and sun in lowland tundra. Late snowmelt shortens the growing season and determines the specific flora composition of snowbed meadows.

Subarctic (or sub-Arctic) – territory from the forest-tundra to the southernmost part of the High Arctic (Matveeva, 1998).

Syntaxon (pl. - syntaxa) – in phytosociology, a conceptual unit of *vegetation* comprising a combination of plant taxa.

Synusia – a sub-community of a *plant community* that is characterized by a particular group of species that share similar ecological requirements and interact with each other.

Vegetation – the assemblage of vascular plants, bryophytes, and lichens, and their associated physical and biological features, that occupy a particular geographic area.

Vegetation type – a unity of *plant communities* similar in ecological requirements and proportions of *plant functional types* of dominant species. Tundra represents a vegetation type dominated by dwarf shrubs, perennial herbs, mosses, and lichens, which is most common within the tundra zone.

Zonal habitats – areas where *vegetation* develops under the prevailing (typical for bioclimatic zone) climate, moderate soil moisture, snow, and soil chemistry. Zonal sites are gently sloping, moderately drained, with fine-grained soils.

Zonal vegetation – *vegetation type* which occurs in *zonal habitats* and is characteristic for a certain bioclimatic (or geobotanical) zone. For example, in the bioclimatic subzone E (CAVM Team, 2003), zonal vegetation is shrub-sedge-moss tundra with dominance of *Betula nana* and low willows (*Salix lanata* and *S. glauca*), whereas in subzone B prostrate shrub-herb-moss tundra communities represent zonal vegetation.

References and major terminological sources consulted:

Allaby, M. (1996) The Concise Oxford Dictionary of Ecology. Oxford University Press, Oxford, UK.

Allaby, M. (ed.) (2006) A Dictionary of Plant Sciences. Revised ed. Oxford University Press, New York, US.

Bliss, L.C., Matveyeva, N.V. (1992) Circumpolar arctic vegetation. In: Chapin, F.S., Jefferies, R.L., Reynolds, J.F., Shaver, G.R., Svoboda, J., Chu, E.W. (eds.) Arctic ecosystems in a changing climate: an ecophysiological perspective, Academic Press, 59-89.

Braun-Blanquet, J. (1964) Pflanzensoziologie: Grundzüge der Vegetationskunde (3rd ed.). Wien: Springer.

Callaghan, T.V., Björn, L.O., Chernov, Y., Chapin, T., Christensen, T., Huntley, B., Ims, R.A., Johansson, M., Jolly, D., Jonasson, S., Matveyeva, N., Panikov, N., Oechel, W., Shaver, G., Henttonen, H. (2004). Effects on the structure of arctic ecosystems in the short- and long-term perspectives. Ambio: a Journal of Human Environment, 33(7), 436-447.

CAVM Team (2003) Circumpolar Arctic vegetation map. Conservation of Arctic Flora and Fauna (CAFF).

Chernov Yu.I., Matveeva N.V. (1997) Arctic Ecosystems in Russia. In: Wielgolaski, F (ed.) Ecosystems of the World 3. Polar and Alpine Tundra, 361-507.

Khitun, O.V., Koroleva, T.M., Chinenko, S.V., Petrovsky, V.V., Pospelova, E.B., Pospelov, I.N., Zverev, A.A. (2016) Applications of local floras for floristic subdivision and monitoring vascular plant diversity in the Russia Arctic. Arctic Science, 2(3), 103–126.

Koroleva, T.M., Zverev, A.A., Katenin, A.E., Petrovsky, V.V., Pospelova, E.B., Rebristaya, O.V., Khitun, O.V., Khodachek, E.A., Chinenko, S.V. (2011) Longitudinal geographical structure of local and regional floras of the Asian Arctic, paper 2. Botanicheskii zhurnal, 96.2, 145-169 (In Russian).

Matveeva, N.V. (1998) Zonation in plant cover of the Arctic. Russian Academy of sciences. Proceedings of Komarov Botanical Institute. 21. 220. (In Russian).

Tolmatchev, A.I. (1974) Introduction to the geography of plants. Leningrad State University Print. (In Russian)

Yurtsev, B.A. (1994) Floristic division of the Arctic. Journal of Vegetation science, 5, 765-776.

Yurtsev, B.A. (1966) Hypoarctic botanical-geographic belt and the origin of its flora. Komarovskie chteniia, 19.93. (In Russian).

Yurtsev, B.A., Kamelin, R.V. (1991) The main concepts and terms of the floristics: the study manual for the course. Perm University Print. (In Russian)

Whittaker, R.H. (1975) Communities and Ecosystems. 2nd Edition, Macmillan Publishing Co., New York, 387 p.

Research conducted in the West Siberian Arctic prior to the present study

While the flora and vegetation of the Yamal and Tazovsky Peninsulas are relatively well studied (e.g., Rebristaya et al., 1989; Telyatnikov, 2003; Rebristaya, 2013), botanical investigations on the Gydansky Peninsula (which occupies more than a half of the West Siberian Arctic), are very limited and have been conducted mainly in its western part (Rebristaya, Khitun, 1994; Khitun, 2002, 2003, 2016; Khitun, Rebristaya, 1998, 2018). Central, and especially eastern, parts of the

peninsula have not been studied since B.N. Gorodkov's works (1916, 1935). Only one locality was studied on the coast of the Yenisei Bay (Kozhevnikov, 1996). Short-term field work in combination with aerial surveillance was carried out while preparing of the map "Vegetation of the West Siberian Plain" with a scale of 1:1500000 (Ilyina et al., 1976). Almost 10 years later these authors published the explanatory text with general characteristics of the region's vegetation types (Ilyina et al., 1985). Although they had only one ground site in the north of Gydansky and one in the north of Yamal, the High-Low Arctic boundary they defined is drawn much further north than in the classical work of Gorodkov (1935).

References:

Gorodkov, B.N. (1916) Botanical-geographical regions division of West Siberian lowland. Ezhegodnik Tobol'skogo gubernskogo muzeia, 27, 1–56. (In Russian).

Gorodkov, B.N. (1935) Vegetation of USSR tundra zone. (In Russian).

Ilyina, I.S., Lapshina, E.I., Makhno, V.D., Romanova, E.A. (1976) Vegetation of the West Siberian Plain. Map. 1:1 500 000. Moskva, GUGK.

Ilyina, I.S., Lapshina, E.I., Lavrenko, N.N., Meltser, L.I., Romanova, E.A., Bogoiavlenskii, B.A., Makhno, V.D. (1985) Rastitel'nyi pokrov Zapadno-Sibirskoi ravniny. (ed. V.V. Vorobev, A.B. Belov), Nauka, Novosibirsk. (In Russian).

Khitun, O.V. (2002) Intralandscape structure of the flora of the Tinikyakha river lower reaches (northern hypoarctic tundra, Gydansy peninsula). Botanicheskii zhurnal, 87(8), 1–24. (In Russian).

Khitun, O.V. (2003) Analysis of the intralandscape structure of the flora of the Khalmeryakha river middle reaches (West Siberian Arctic). Botanicheskii zhurnal, 88(10), 9–30. (In Russian).

Khitun, O.V. (2016) Intralandscape differentiation of the local flora in the central part of the Gydansky Peninsula (West Siberian Arctic). Czech Polar Reports, 6(2), 191-209.

Khitun, O.V., Rebristaya, O. V. (1998) Vegetation and ecotopical structure of the flora of cape Khonorasale surroundings (Gydansky peninsula, the arctic tundra subzone). Botanicheskii zhurnal, 83(12), 21–37. (In Russian).

Khitun O.V., Rebristaia O. V. (2018) Local flora of Cape Matuisale surroundings – the only well studied by botanists part of the Gydan Nature Reserve. Ekologiia i geografiia rastenii i rastitel'nykh soobshchestv, 997–1000. (In Russian).

Kozhevnikov, Yu.P. (1996) Floristical features of Enisenian forest-tundra. Botanicheskii zhurnal, 81(2), 68–82. (In Russian).

Rebristaya, O.V., Tvorogov, V.A., Khitun, O.V. (1989) Flora of Tazovsky peninsula (North of Western Siberia). Botanicheskii zhurnal, 74(1), 22–35. (In Russian).

Rebristaya, O.V., Khitun, O.V. (1994) Flora of the vascular plants in the lower reaches of the Chugoriyakha River (south-western part of the Gydan Peninsula, West Siberian Arctic). Botanicheskii zhurnal. 79.8, 68–77. (In Russian).

Rebristaya, O.V. (2013) Flora of the Yamal Peninsula. Modern State and History of the Formation. SPb, Publish.house of ETU, 312. (In Russian).

Telyatnikov, M.Yu. (2003) Vegetation of typical tundra of the Yamal Peninsula. Novosibirsk. Nauka, 123. (In Russian).
