# Supplementary material for: The High–Low Arctic boundary: How is it determined and where is it located?
Source: Ecol Evol. 2023 Sep 28;13(10):e10545. doi: 10.1002/ece3.10545 (PMC10539046; doi:10.1002/ece3.10545)
Supplement: Supplementary file 2 — Appendix S2 [file ECE3-13-e10545-s001.pdf]

Appendix 2. Full set of the model layers.

Table 2.1. Main layers - CHELSA set.

N in the dataset	Shortname	Longname	Unit	Description
**1**	bio1	mean annual air temperature	°C/10	mean annual daily mean air temperatures averaged over 1 year
**2**	bio2	mean diurnal air temperature range	°C/10	mean diurnal range of temperatures averaged over 1 year
**3**	bio3	isothermality	°C/10	ratio of diurnal variation to annual variation in temperatures
**4**	bio4	temperature seasonality	°C/10	standard deviation of the monthly mean temperatures
**5**	bio5	mean daily maximum air temperature of the warmest month	°C/10	The highest temperature of any monthly daily mean maximum temperature
**6**	bio6	mean daily minimum air temperature of the coldest month	°C/10	The lowest temperature of any monthly daily mean maximum temperature
**7**	bio7	annual range of air temperature	°C/10	The difference between the Maximum Temperature of Warmest month and the Minimum Temperature of Coldest month
**8**	bio8	mean daily mean air temperatures of the wettest quarter	°C/10	The wettest quarter of the year is determined (to the nearest month)
**9**	bio9	mean daily mean air temperatures of the driest quarter	°C/10	The driest quarter of the year is determined (to the nearest month)

**10**	bio10	mean daily mean air temperatures of the warmest quarter	°C/10	The warmest quarter of the year is determined (to the nearest month)
**11**	bio11	mean daily mean air temperatures of the coldest quarte	°C/10	The coldest quarter of the year is determined (to the nearest month)
**12**	bio12	annual precipitation amount	kg m^{-2}	Accumulated precipitation amount over 1 year
**13**	bio13	precipitation amount of the wettest month	kg m^{-2}	The precipitation of the wettest month.
**14**	bio14	precipitation amount of the driest month	kg m^{-2}	The precipitation of the driest month.
**15**	bio15	precipitation seasonality	kg m^{-2}	The Coefficient of Variation is the standard deviation of the monthly precipitation estimates expressed as a percentage of the mean of those estimates (i.e. the annual mean)
**16**	bio16	mean monthly precipitation amount of the wettest quarter	kg m^{-2}	The wettest quarter of the year is determined (to the nearest month)
**17**	bio17	mean monthly precipitation amount of the driest quarter	kg m^{-2}	The driest quarter of the year is determined (to the nearest month)
**18**	bio18	mean monthly precipitation amount of the warmest quarter	kg m^{-2}	The warmest quarter of the year is determined (to the nearest month)
**19**	bio19	mean monthly precipitation amount of the coldest quarter	kg m^{-2}	The coldest quarter of the year is determined (to the nearest month)

Table 2.2. Additional layers - exChelsa – extended bioclim.

N in the dataset	Shortname	Longname	Unit	Explanation
**20**	gsl	growing season length TREELIM	number of days	Length of the growing season
**21**	gst	Mean temperature of the growing season TREELIM	°C/10	Mean temperature of all growing season days based on TREELIM (https://doi.org/10.1007/s00035- 014-0124-0)
**22**	gdd10	Growing degree days heat sum above 10°C	°C	heat sum of all days above the 10°C temperature accumulated over 1 year.
**23**	fcf	Frost change frequency	count	Number of events in which tmin or tmax go above, or below 0°C
**24**	nfd	Number of frost days	count	Number of days at which tmin < 0°C
**25**	swd	snow cover days	count	Number of days with snowcover
**26**	swe	snow water equivalent	kg m^{-2}	Amount of luquid water if snow is melted
**27**	gsp	Accumulated precipiation amount on days above 5°C	kg m^{-2}	precipitation sum accumulated on all days above the threshold temperature over 1 year
**28**	gdd5	Growing degree days heat sum above 5°C	°C	heat sum of all days above the 5°C temperature accumulated over 1 year.
**29**	gsp2	Accumulated precipiation	kg m^{-2}	The amount of precipitation during the growing season

		amount on the growing season		(days with temperatures above 0.9 ° C, without snow and soil moisture > 2 kg/m2)
**30**	shc	Selyaninov's Hydrothermic Coefficient	(kg/m^2/10)/Celsius	The amount of precipitation for the period when the air temperature is above + 5 ° C is divided by the sum of the average daily temperatures for the same period, reduced to its tenth part
**31**	rh	relative humidity	%	Daily mean near surface relative humidity averaged over 1 month

Table 2.3. Additional layers – MODIS data and EarthEnv data.

**N in the dataset**	**Shortname**	**Longname**	**Unit**	**Explanation**
**32**	modcf	intra-annual variability of cloudiness	number of days	intra-annual cloud variability<br>(EarthEnv)
**33**	mod7	mean land surface temperature in July	K	the temperature of the land surface in July<br>(MODIS)
**34**	mod1	mean land surface temperature in January	K	the temperature of the land surface in January<br>(MODIS)
