# Supplementary material for: The High–Low Arctic boundary: How is it determined and where is it located?
Source: Ecol Evol. 2023 Sep 28;13(10):e10545. doi: 10.1002/ece3.10545 (PMC10539046; doi:10.1002/ece3.10545)
Supplement: Supplementary file 3 — Appendix S3 [file ECE3-13-e10545-s005.pdf]

Appendix 3. Correlation matrix of model layers and predictor layers subsets used for species distribution modelling.

Appendix 3a. Correlation matrix of the full set of model layers.

The correlation absolute values over 0.75 are marked with red. See Appendix 1 for description of the short names of the model layers.

	bio1	bio2	bio3	bio4	bio5	bio6	bio7	bio8	bio9	bio 10	bio 11	bio 12	bio 13	bio 14	bio 15	bio 16	bio 17	bio 18	bio 19	fcf	gdd5	gsp	gsp2	nfd	shc	swd	gdd 10	gsl	gst	mod cf	mod 7	mod 1	rh
bio1	1,00	0,55	0,64	0,13	0,77	0,77	0,33	0,80	-0,51	0,83	0,85	0,91	0,95	0,16	0,77	0,96	0,47	0,94	0,00	0,00	0,01	0,96	0,01	0,00	0,01	0,00	0,69	0,00	0,01	-0,51	0,61	0,13	-0,88
bio2	0,55	1,00	0,79	0,78	0,90	-0,07	0,92	0,75	-0,51	0,85	0,07	0,69	0,65	0,26	0,48	0,66	0,46	0,67	0,09	0,00	0,04	0,71	0,04	0,00	0,04	0,00	0,70	0,00	0,03	-0,54	0,61	-0,11	-0,64
bio3	0,64	0,79	1,00	0,25	0,66	0,29	0,48	0,52	-0,44	0,63	0,42	0,59	0,63	0,12	0,57	0,64	0,30	0,67	-0,07	0,00	0,05	0,70	0,04	0,00	0,04	0,00	0,49	0,00	0,02	-0,51	0,42	0,20	-0,66
bio4	0,13	0,78	0,25	1,00	0,72	-0,50	0,96	0,60	-0,31	0,66	-0,40	0,41	0,32	0,26	0,11	0,31	0,38	0,31	0,21	0,00	0,03	0,35	0,03	0,00	0,03	0,00	0,56	0,00	0,02	-0,30	0,49	-0,39	-0,28
bio5	0,77	0,90	0,66	0,72	1,00	0,22	0,85	0,93	-0,58	0,99	0,34	0,88	0,86	0,29	0,64	0,86	0,57	0,85	0,10	0,00	0,03	0,88	0,03	0,00	0,02	0,00	0,83	0,00	0,02	-0,57	0,74	-0,12	-0,78
bio6	0,77	-0,07	0,29	-0,50	0,22	1,00	-0,33	0,35	-0,23	0,31	0,98	0,53	0,63	-0,01	0,60	0,63	0,19	0,62	-0,12	0,00	-0,01	0,60	-0,01	0,00	-0,01	0,00	0,26	0,00	-0,01	-0,23	0,24	0,31	-0,57
bio7	0,33	0,92	0,48	0,96	0,85	-0,33	1,00	0,71	-0,43	0,79	-0,21	0,56	0,49	0,28	0,29	0,49	0,45	0,49	0,16	0,00	0,04	0,53	0,03	0,00	0,03	0,00	0,66	0,00	0,03	-0,42	0,58	-0,29	-0,45
bio8	0,80	0,75	0,52	0,60	0,93	0,35	0,71	1,00	-0,60	0,93	0,44	0,86	0,87	0,21	0,68	0,86	0,52	0,85	0,00	0,00	0,02	0,86	0,01	0,00	0,01	0,00	0,80	0,00	0,02	-0,49	0,72	-0,12	-0,76
bio9	-0,51	-0,51	-0,44	-0,31	-0,58	-0,23	-0,43	-0,60	1,00	-0,54	-0,29	-0,44	-0,59	0,13	-0,72	-0,57	-0,11	-0,55	0,50	0,00	-0,01	-0,55	-0,01	0,00	-0,01	0,00	-0,47	0,00	-0,01	0,33	-0,46	-0,02	0,45
bio 10	0,83	0,85	0,63	0,66	0,99	0,31	0,79	0,93	-0,54	1,00	0,42	0,92	0,89	0,27	0,64	0,89	0,57	0,88	0,13	0,00	0,03	0,92	0,02	0,00	0,02	0,00	0,84	0,00	0,02	-0,56	0,74	-0,12	-0,82
bio 11	0,85	0,07	0,42	-0,40	0,34	0,98	-0,21	0,44	-0,29	0,42	1,00	0,63	0,71	0,01	0,65	0,72	0,24	0,71	-0,10	0,00	-0,01	0,70	-0,01	0,00	-0,01	0,00	0,35	0,00	0,00	-0,30	0,31	0,30	-0,67
bio 12	0,91	0,69	0,59	0,41	0,88	0,53	0,56	0,86	-0,44	0,92	0,63	1,00	0,95	0,40	0,61	0,96	0,70	0,94	0,26	0,00	0,02	0,96	0,01	0,00	0,01	0,00	0,77	0,00	0,02	-0,55	0,68	0,00	-0,86
bio 13	0,95	0,65	0,63	0,32	0,86	0,63	0,49	0,87	-0,59	0,89	0,71	0,95	1,00	0,17	0,82	1,00	0,49	0,98	-0,01	0,00	0,02	0,98	0,01	0,00	0,01	0,00	0,75	0,00	0,02	-0,55	0,68	0,07	-0,86
bio 14	0,16	0,26	0,12	0,26	0,29	-0,01	0,28	0,21	0,13	0,27	0,01	0,40	0,17	1,00	-0,29	0,19	0,93	0,20	0,72	0,00	0,01	0,19	0,01	0,00	0,01	0,00	0,22	0,00	0,00	-0,21	0,21	-0,05	-0,21
bio 15	0,77	0,48	0,57	0,11	0,64	0,60	0,29	0,68	-0,72	0,64	0,65	0,61	0,82	-0,29	1,00	0,80	0,00	0,79	-0,50	0,00	0,02	0,77	0,01	0,00	0,01	0,00	0,54	0,00	0,01	-0,42	0,52	0,15	-0,66
bio 16	0,96	0,66	0,64	0,31	0,86	0,63	0,49	0,86	-0,57	0,89	0,72	0,96	1,00	0,19	0,80	1,00	0,51	0,98	0,02	0,00	0,02	0,99	0,01	0,00	0,01	0,00	0,75	0,00	0,02	-0,55	0,67	0,07	-0,87

	bio1	bio2	bio3	bio4	bio5	bio6	bio7	bio8	bio9	bio 10	bio 11	bio 12	bio 13	bio 14	bio 15	bio 16	bio 17	bio 18	bio 19	fcf	gdd5	gsp	gsp2	nfd	shc	swd	gdd 10	gsl	gst	mod cf	mod 7	mod 1	rh
bio 17	0,47	0,46	0,30	0,38	0,57	0,19	0,45	0,52	-0,11	0,57	0,24	0,70	0,49	0,93	0,00	0,51	1,00	0,51	0,64	0,00	0,01	0,51	0,01	0,00	0,01	0,00	0,48	0,00	0,01	-0,36	0,43	-0,06	-0,49
bio 18	0,94	0,67	0,67	0,31	0,85	0,62	0,49	0,85	-0,55	0,88	0,71	0,94	0,98	0,20	0,79	0,98	0,51	1,00	0,03	0,00	0,02	0,98	0,01	0,00	0,01	0,00	0,74	0,00	0,02	-0,56	0,67	0,08	-0,88
bio 19	0,00	0,09	-0,07	0,21	0,10	-0,12	0,16	0,00	0,50	0,13	-0,10	0,26	-0,01	0,72	-0,50	0,02	0,64	0,03	1,00	0,00	0,00	0,05	0,00	0,00	0,00	0,00	0,10	0,00	0,00	-0,07	0,05	-0,10	-0,10
fcf	0,00	0,00	0,00	0,00	0,00	0,00	0,00	0,00	0,00	0,00	0,00	0,00	0,00	0,00	0,00	0,00	0,00	0,00	0,00	1,00	0,99	0,00	0,99	1,00	0,99	1,00	0,46	1,00	0,95	0,53	-0,03	0,03	-0,26
gdd5	0,01	0,04	0,05	0,03	0,03	-0,01	0,04	0,02	-0,01	0,03	-0,01	0,02	0,02	0,01	0,02	0,02	0,01	0,02	0,00	0,99	1,00	0,02	1,00	0,99	1,00	0,99	0,47	0,99	0,94	0,51	-0,01	0,03	-0,27
gsp	0,96	0,71	0,70	0,35	0,88	0,60	0,53	0,86	-0,55	0,92	0,70	0,96	0,98	0,19	0,77	0,99	0,51	0,98	0,05	0,00	0,02	1,00	0,02	0,00	0,01	0,00	0,76	0,00	0,02	-0,57	0,67	0,06	-0,89
gsp2	0,01	0,04	0,04	0,03	0,03	-0,01	0,03	0,01	-0,01	0,02	-0,01	0,01	0,01	0,01	0,01	0,01	0,01	0,01	0,00	0,99	1,00	0,02	1,00	0,99	1,00	0,99	0,47	0,99	0,94	0,51	-0,01	0,03	-0,27
nfd	0,00	0,00	0,00	0,00	0,00	0,00	0,00	0,00	0,00	0,00	0,00	0,00	0,00	0,00	0,00	0,00	0,00	0,00	0,00	1,00	0,99	0,00	0,99	1,00	0,99	1,00	0,46	1,00	0,95	0,53	-0,03	0,03	-0,26
shc	0,01	0,04	0,04	0,03	0,02	-0,01	0,03	0,01	-0,01	0,02	-0,01	0,01	0,01	0,01	0,01	0,01	0,01	0,01	0,00	0,99	1,00	0,01	1,00	0,99	1,00	0,99	0,47	0,99	0,94	0,51	-0,01	0,03	-0,27
swd	0,00	0,00	0,00	0,00	0,00	0,00	0,00	0,00	0,00	0,00	0,00	0,00	0,00	0,00	0,00	0,00	0,00	0,00	0,00	1,00	0,99	0,00	0,99	1,00	0,99	1,00	0,46	1,00	0,95	0,53	-0,03	0,03	-0,26
gdd 10	0,69	0,70	0,49	0,56	0,83	0,26	0,66	0,80	-0,47	0,84	0,35	0,77	0,75	0,22	0,54	0,75	0,48	0,74	0,10	0,46	0,47	0,76	0,47	0,46	0,47	0,46	1,00	0,46	0,45	-0,20	0,61	-0,10	-0,85
gsl	0,00	0,00	0,00	0,00	0,00	0,00	0,00	0,00	0,00	0,00	0,00	0,00	0,00	0,00	0,00	0,00	0,00	0,00	0,00	1,00	0,99	0,00	0,99	1,00	0,99	1,00	0,46	1,00	0,95	0,53	-0,02	0,03	-0,26
gst	0,01	0,03	0,02	0,02	0,02	-0,01	0,03	0,02	-0,01	0,02	0,00	0,02	0,02	0,00	0,01	0,02	0,01	0,02	0,00	0,95	0,94	0,02	0,94	0,95	0,94	0,95	0,45	0,95	1,00	0,52	0,00	0,01	-0,25
mod cf	-0,51	-0,54	-0,51	-0,30	-0,57	-0,23	-0,42	-0,49	0,33	-0,56	-0,30	-0,55	-0,55	-0,21	-0,42	-0,55	-0,36	-0,56	-0,07	0,53	0,51	-0,57	0,51	0,53	0,51	0,53	-0,20	0,53	0,52	1,00	-0,43	-0,04	0,38
mod 7	0,61	0,61	0,42	0,49	0,74	0,24	0,58	0,72	-0,46	0,74	0,31	0,68	0,68	0,21	0,52	0,67	0,43	0,67	0,05	-0,03	-0,01	0,67	-0,01	-0,03	-0,01	-0,03	0,61	-0,02	0,00	-0,43	1,00	-0,19	-0,58
mod 1	0,13	-0,11	0,20	-0,39	-0,12	0,31	-0,29	-0,12	-0,02	-0,12	0,30	0,00	0,07	-0,05	0,15	0,07	-0,06	0,08	-0,10	0,03	0,03	0,06	0,03	0,03	0,03	0,03	-0,10	0,03	0,01	-0,04	-0,19	1,00	-0,06
rh	-0,88	-0,64	-0,66	-0,28	-0,78	-0,57	-0,45	-0,76	0,45	-0,82	-0,67	-0,86	-0,86	-0,21	-0,66	-0,87	-0,49	-0,88	-0,10	-0,26	-0,27	-0,89	-0,27	-0,26	-0,27	-0,26	-0,85	-0,26	-0,25	0,38	-0,58	-0,06	1,00

Appendix 3b. Predictor layers subsets used for species distribution modelling.

Subset 1 (Automatic) is the result of automatic selection of layers with a threshold correlation value of 0.75 and with the use of the "Remove highly correlated variables" algorithm from the SDMtoolbox package of ArcGIS:

1. modcf - intra-annual variability of cloudiness
2. mod1 - mean land surface temperature in January
3. mod7 - mean land surface temperature in July
4. gdd5 - growing degree days heat sum above 5°C
5. bio19 - mean monthly precipitation of the coldest season
6. bio14 - precipitation in the driest month
7. bio9 - mean daily air temperatures of the driest season
8. bio2 - mean daily range of air temperatures
9. bio1 - mean annual air temperature.

Subset 2 (PCA) was generated by principal component transformations of layers in SAGA GIS software and included 19 layers

Subset 3 (Expert) is the result of expert selection of layers with a threshold correlation value of 0.75:

1. bio1 - mean annual air temperature
2. bio2 - mean daily range of air temperatures
3. bio9 - mean daily air temperatures of the driest season
4. bio14 - precipitation during the driest season
5. bio19 - average monthly precipitation of the coldest season
6. fcf - frequency of frosts changing
7. gdd10 - growing degree days heat sum above 10°C
8. modcf - intra-annual variability of cloudiness
9. mod1 - mean land surface temperature in January
10. mod7 - mean land surface temperature in July
