# Supplementary material for: The High–Low Arctic boundary: How is it determined and where is it located?
Source: Ecol Evol. 2023 Sep 28;13(10):e10545. doi: 10.1002/ece3.10545 (PMC10539046; doi:10.1002/ece3.10545)

Appendix 4. Results of the species distribution modelling.

The assessment of the models' quality was based on the AUC criterion calculated in MaxEnt (area under receiver operating characteristic curve). All the models obtained meet the formal quality requirements (AUC ranged from 7.36 to 9.2) and could be used to define the northern boundary of the species ranges. Final models were limited by the threshold value of "Maximum test sensitivity plus specificity" obtained in MaxEnt.
This probability indicator shows that randomly selected occurrence records are better predicted than randomly selected background points (Fielding, Bell, 1997). A random distribution of the modelling results corresponds to the value of AUC=0.5. Based on the AUC value, the quality of the modelling is classified as (Araújo et al., 2005): 0.9–1 - "excellent", 0.8–0.9 - "good", 0.7–0.8 - "satisfactory", 0.6–0.7 - "poor", <0.6 - "failed".

Table 4.1. AUC values for different sets of model layers

	Model layers				
**Species under modelling**		**Full set**	**Subset 1 (Automatic)**	**Subset 2 (PCA)**	**Subset 3 (Expert)**
	Betula nana	0.821	0.766	0.858	0.753
	Salix lanata	0.870	0.816	0.920	0.819
	Empetrum nigrum	0.863	0.834	0.916	0.841
	Rhododendron tomentosum subsp. *decumbens* and *Rh. tomentosum* subsp. *tomentosum*	0.825	0.769	0.896	0.753
	Rubus chamaemorus	0.818	0.765	0.881	0.763
	Vaccinium uliginosum subsp. *microphyllum*	0.798	0.745	0.873	0.736
	Arctous alpina	0.863	0.800	0.919	0.806

Betula nana

Full set

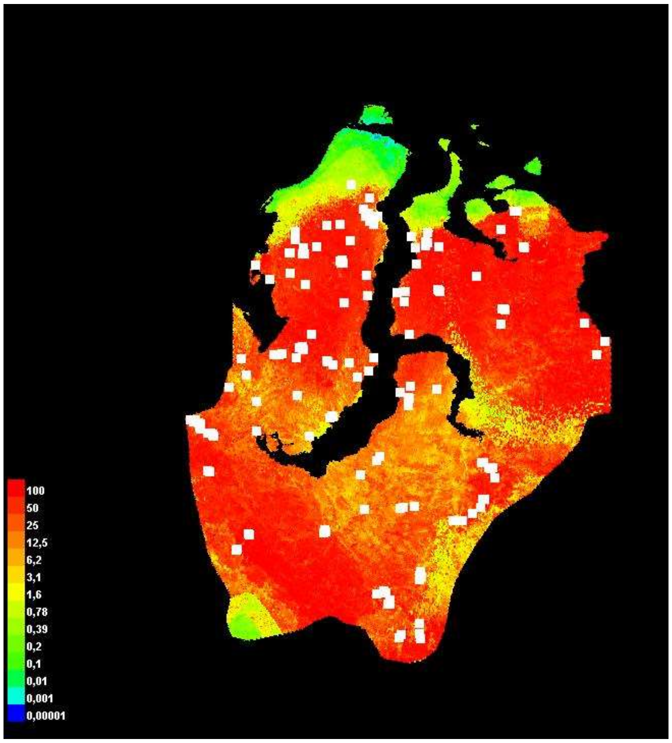

Subset 1 (Automatic)

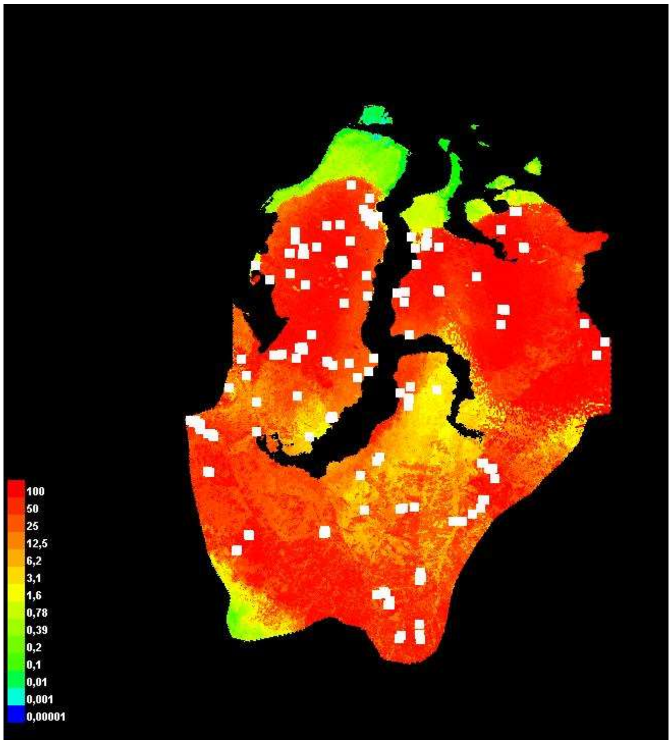

Subset 2 (PCA)

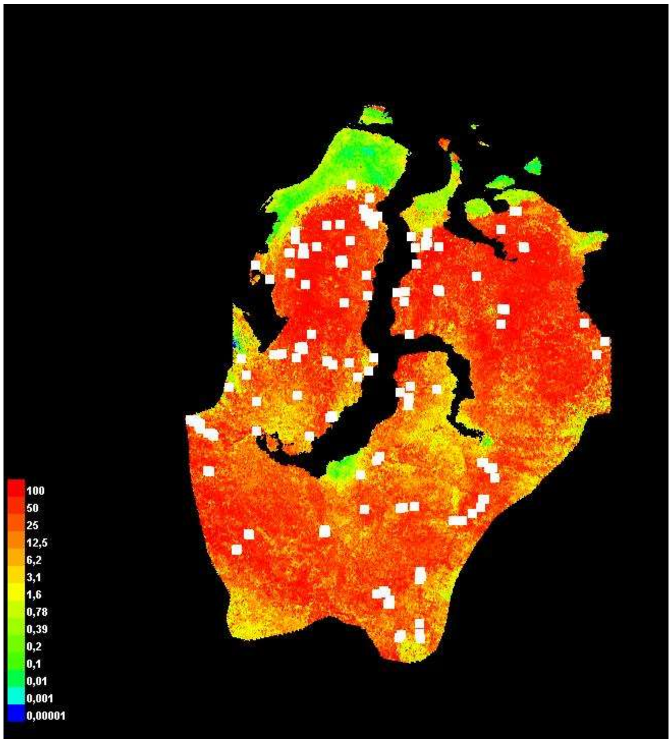

Subset 3 (Expert)

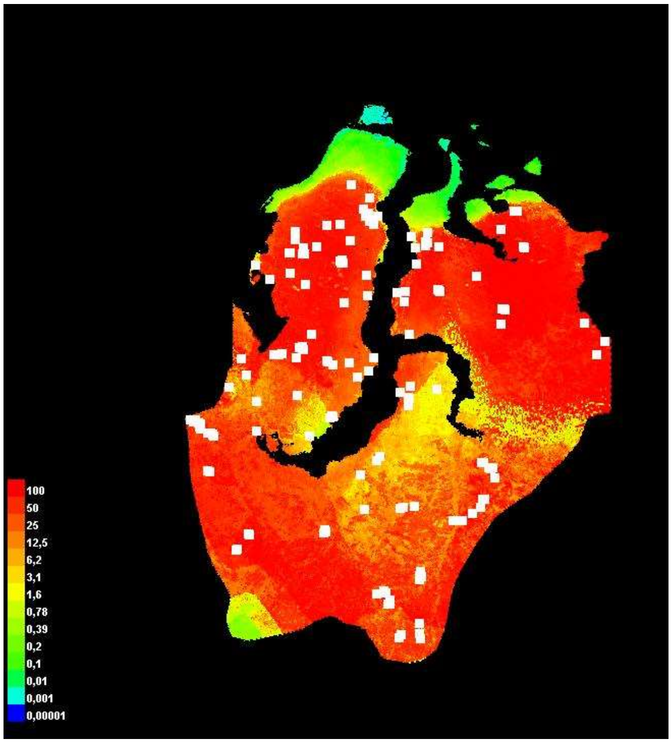

Salix lanata

Full set

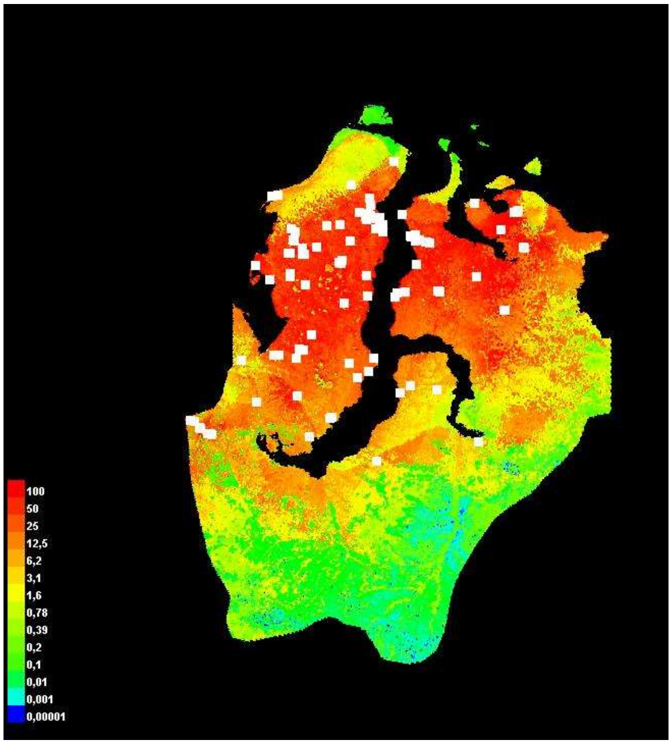

Subset 1 (Automatic)

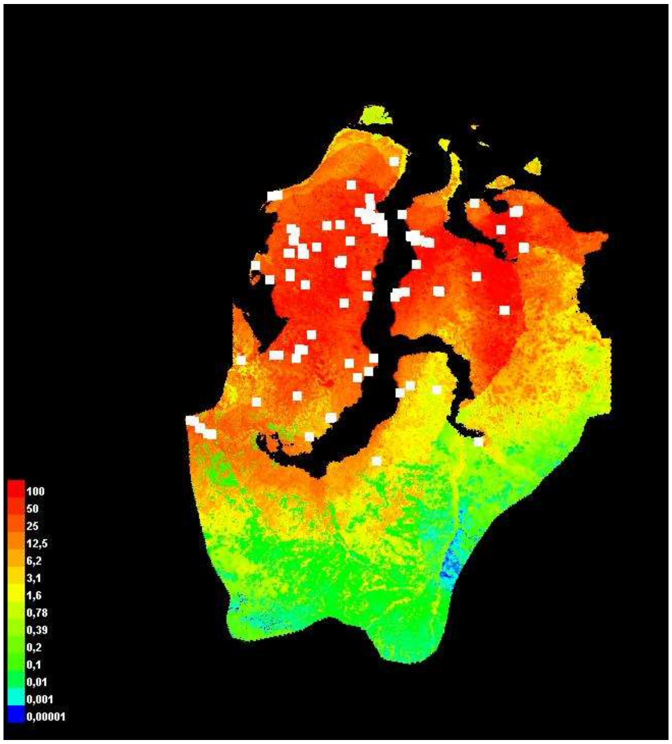

Subset 2 (PCA)

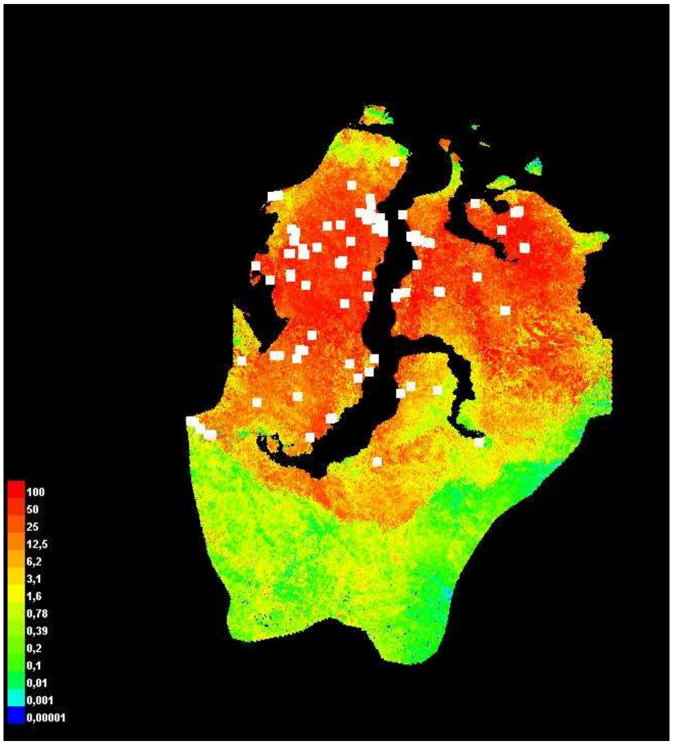

Subset 3 (Expert)

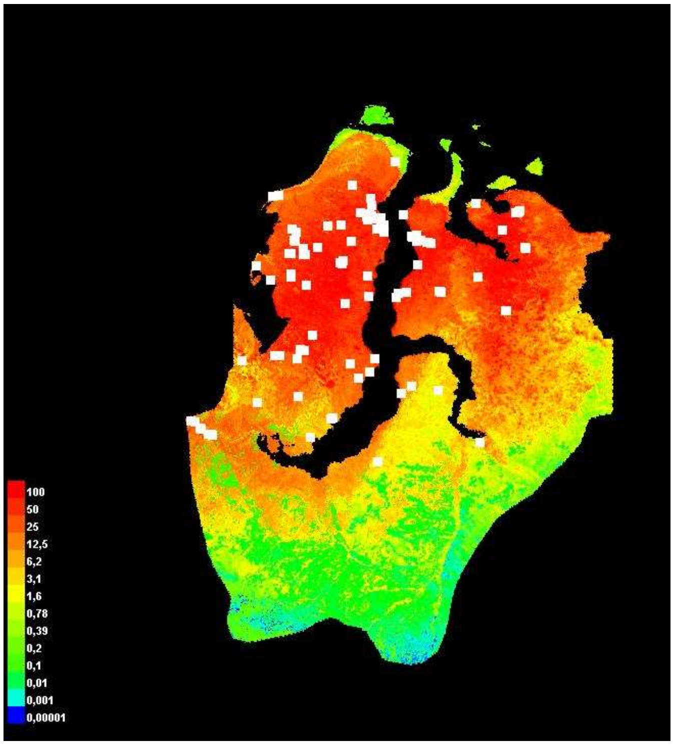

Empetrum nigrum

Full set

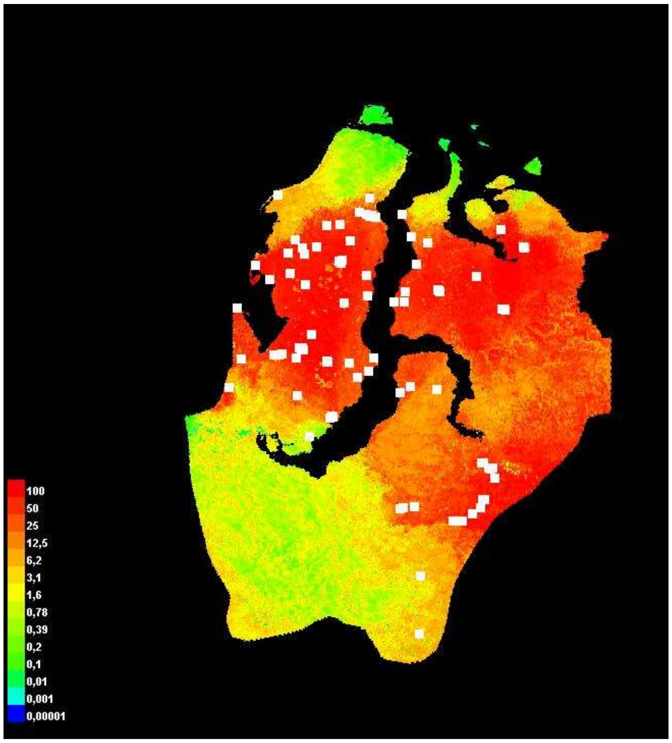

Subset 1 (Automatic)

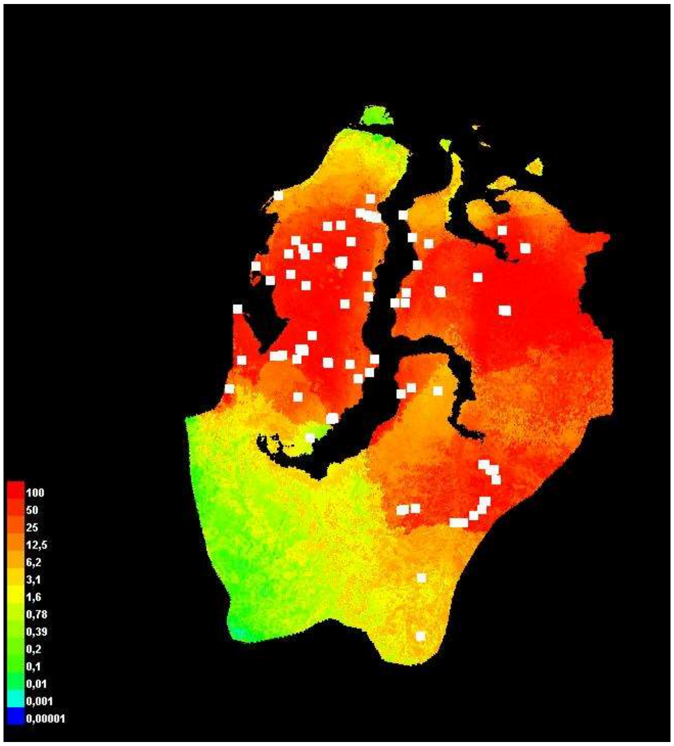

Subset 2 (PCA)

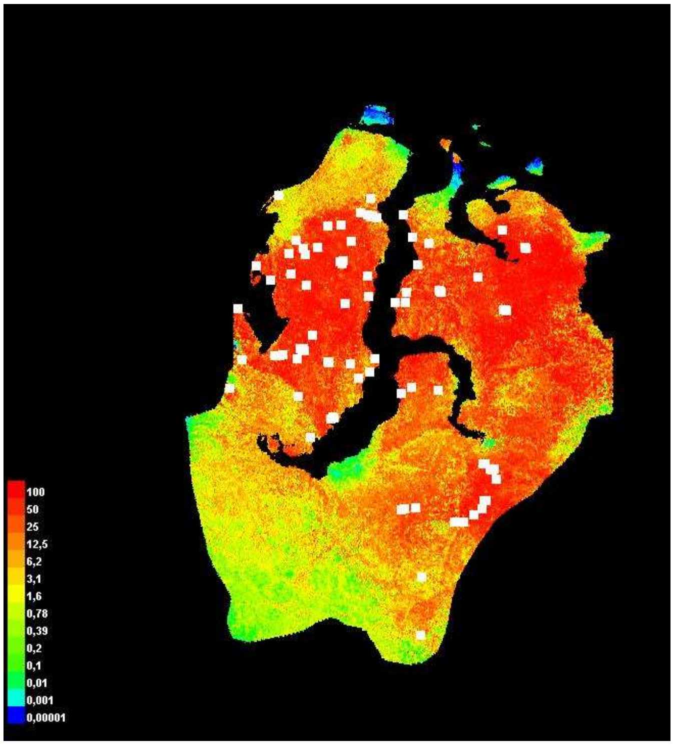

Subset 3 (Expert)

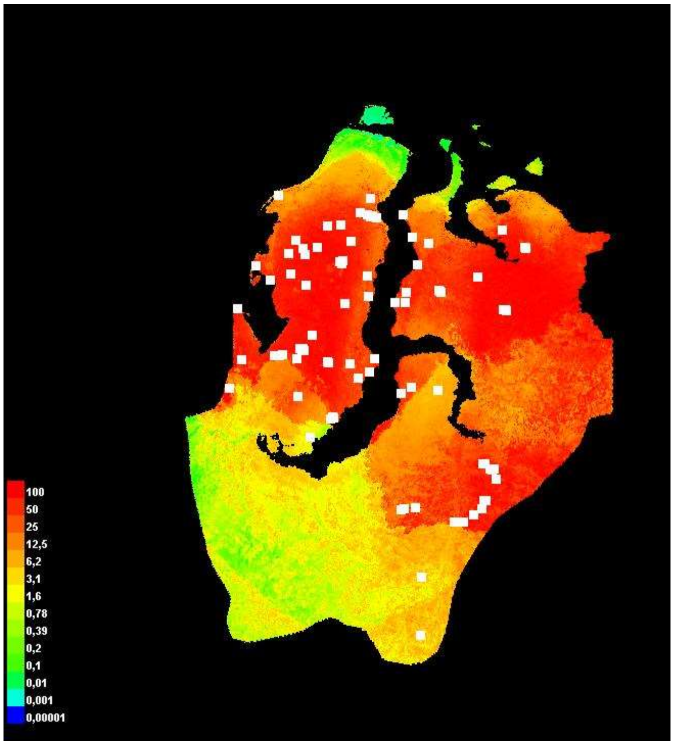

Rhododendron tomentosum subsp. *decumbens* and *Rh. tomentosum* subsp. *tomentosum*

Full set

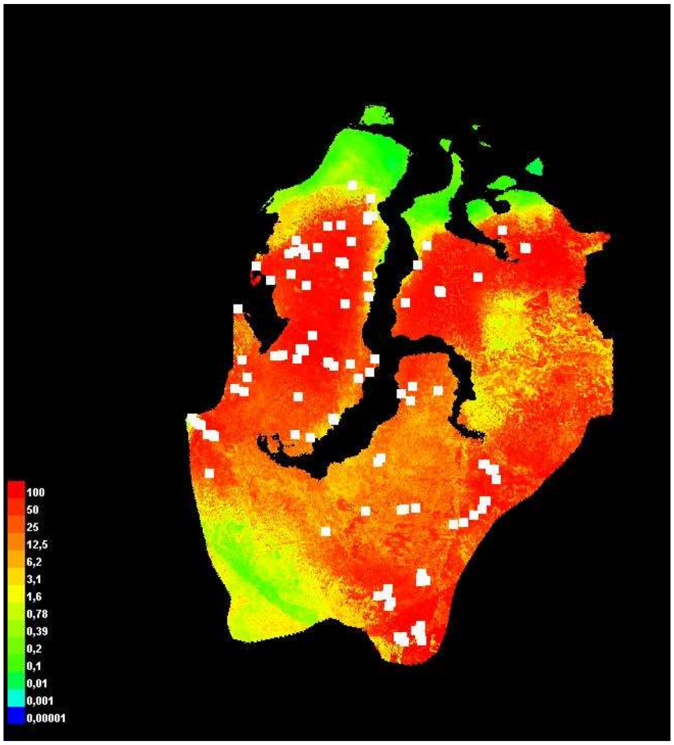

Subset 1 (Automatic)

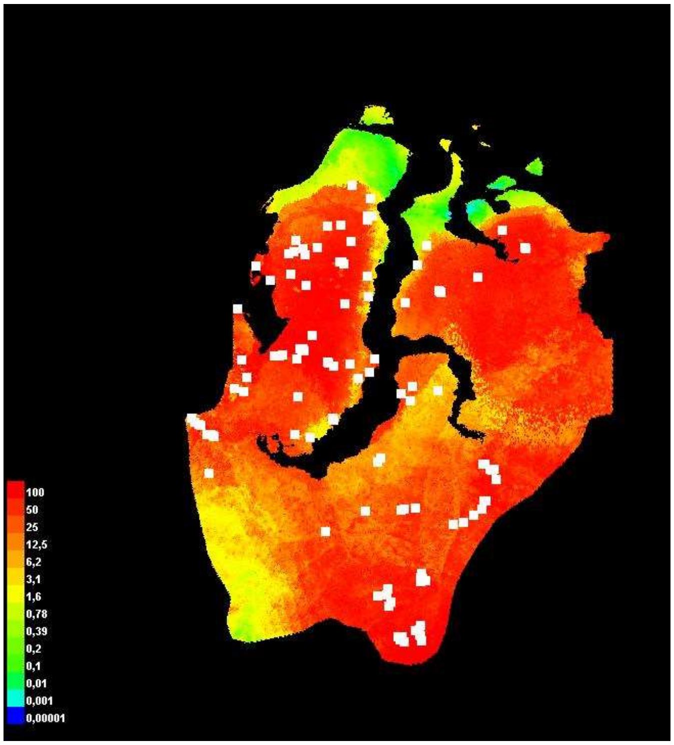

Subset 2 (PCA)

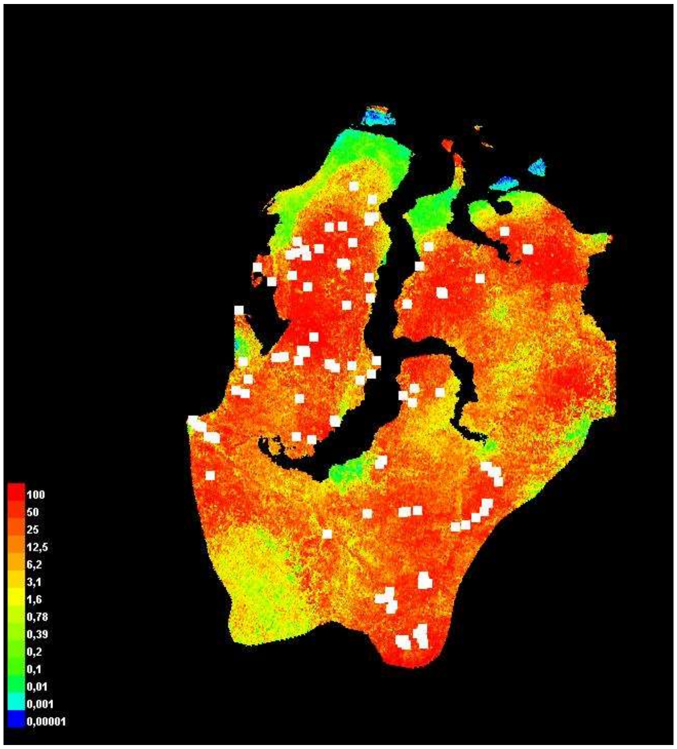

Subset 3 (Expert)

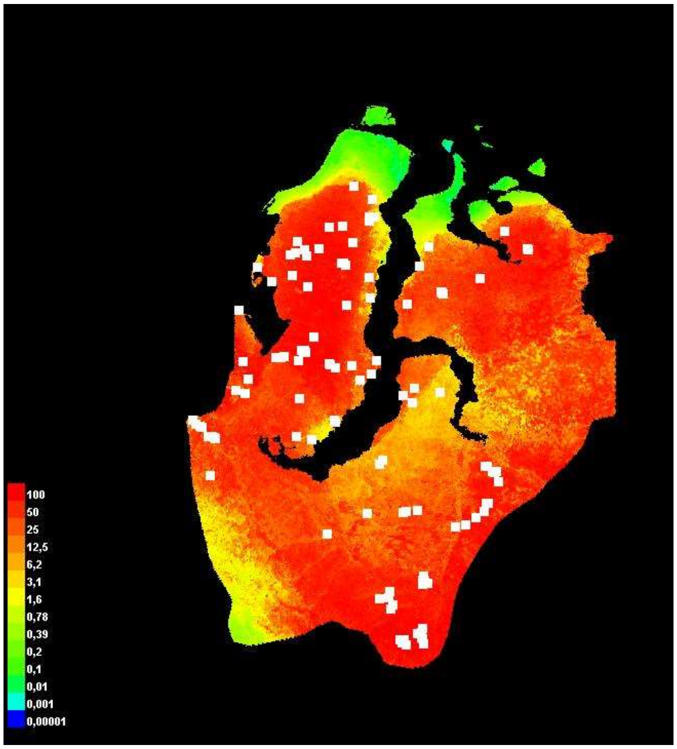

Rubus chamaemorus

Full set

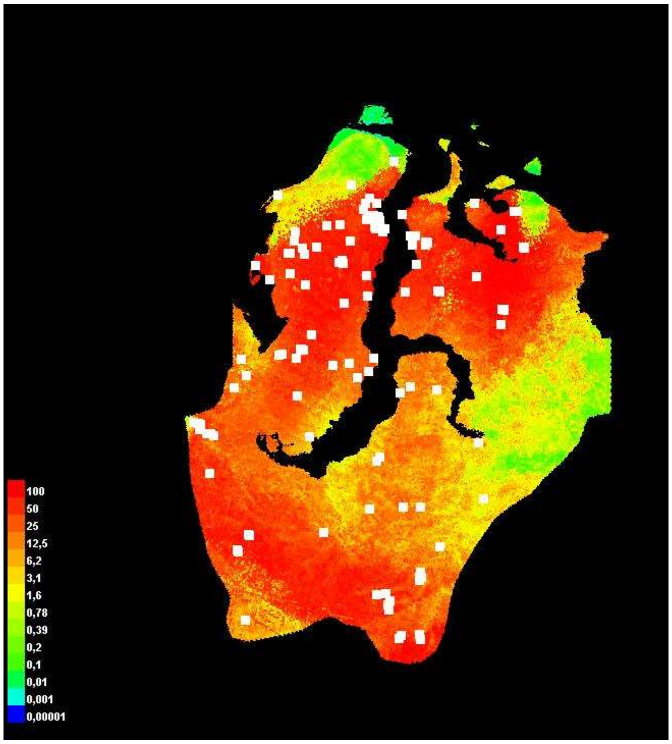

Subset 1 (Automatic)

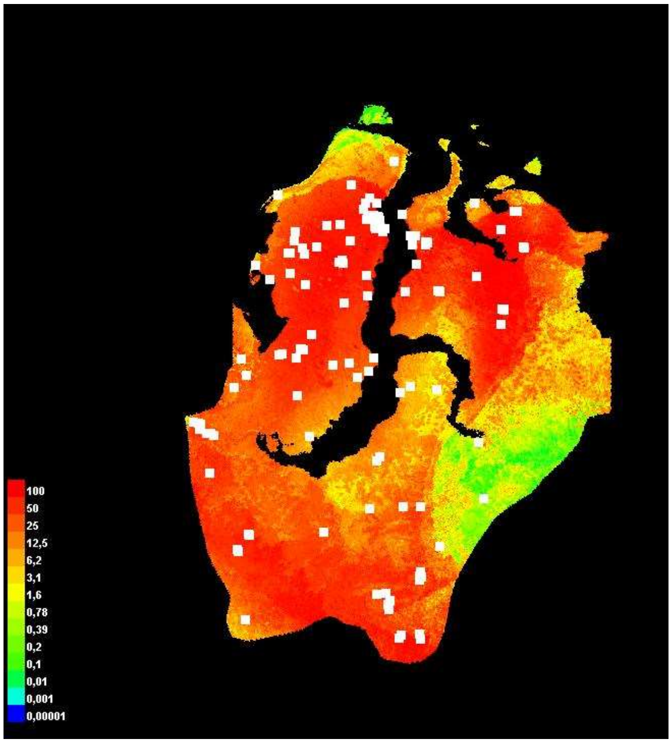

Subset 2 (PCA)

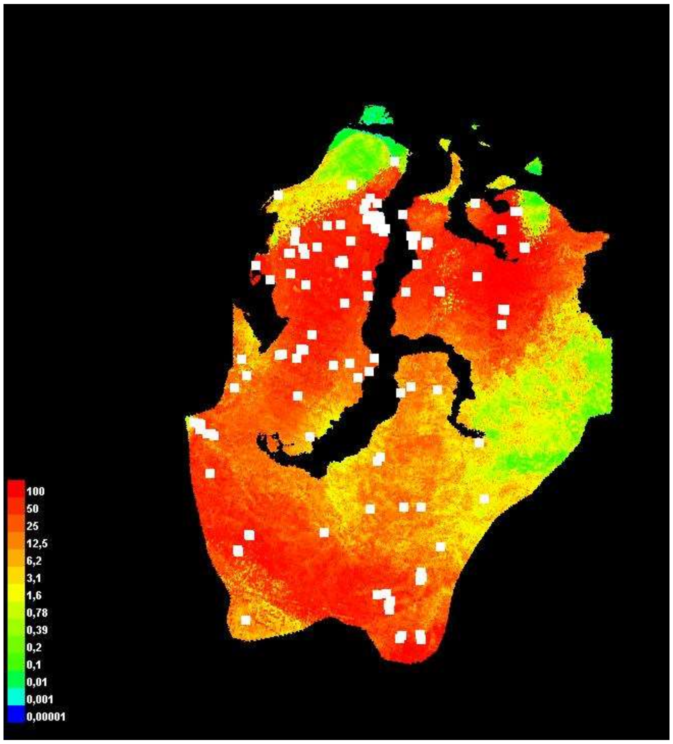

Subset 3 (Expert)

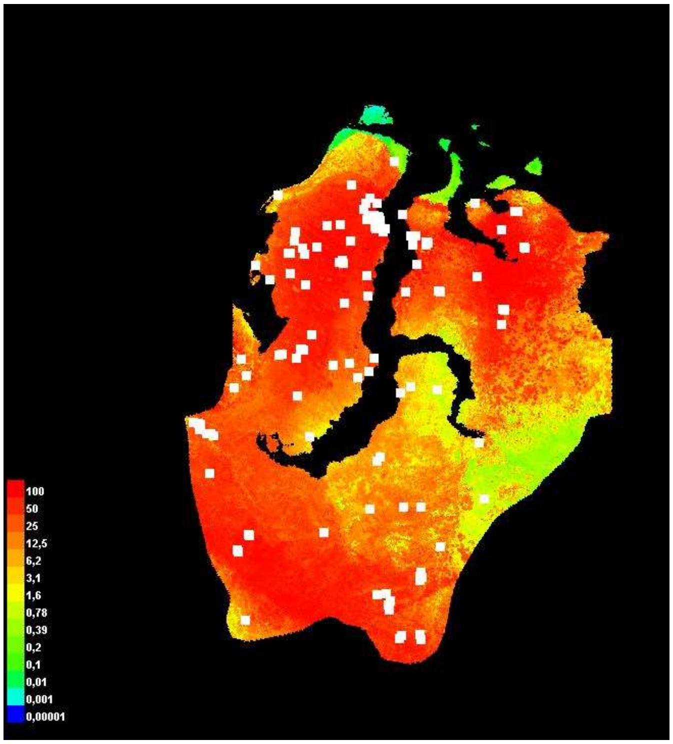

Vaccinium uliginosum subsp. *microphyllum*

Full set

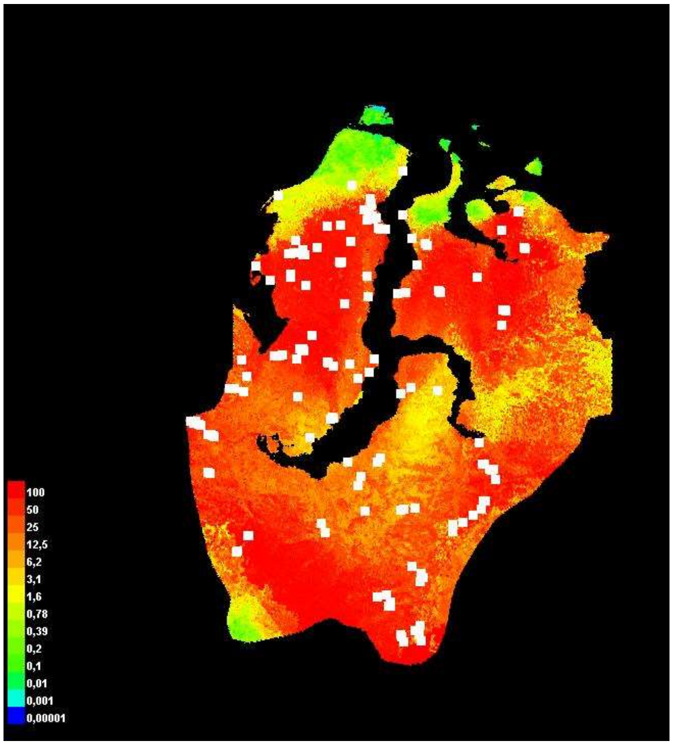

Subset 1 (Automatic)

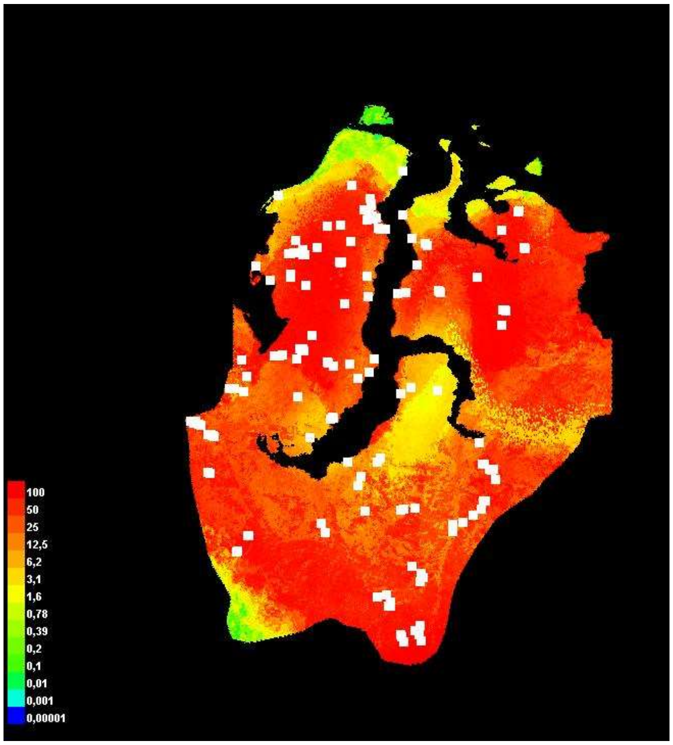

Subset 2 (PCA)

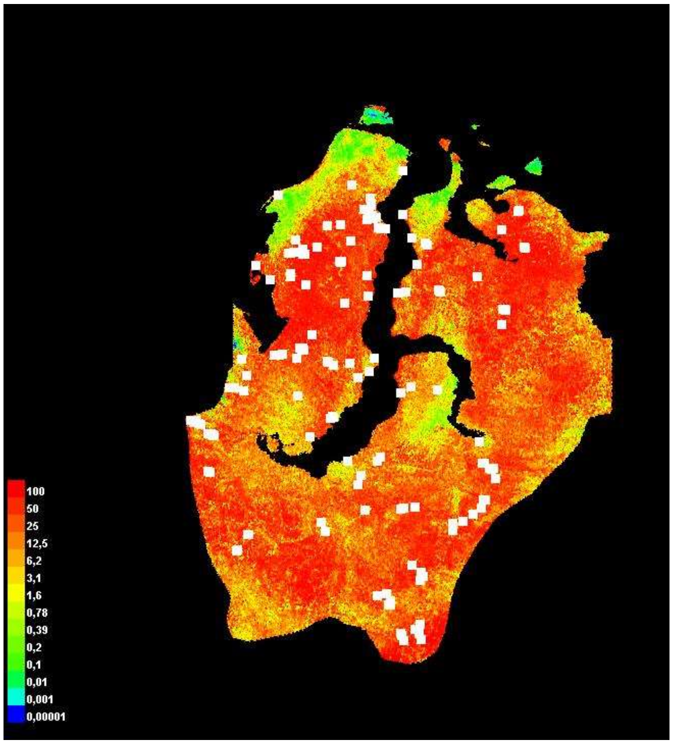

Subset 3 (Expert)

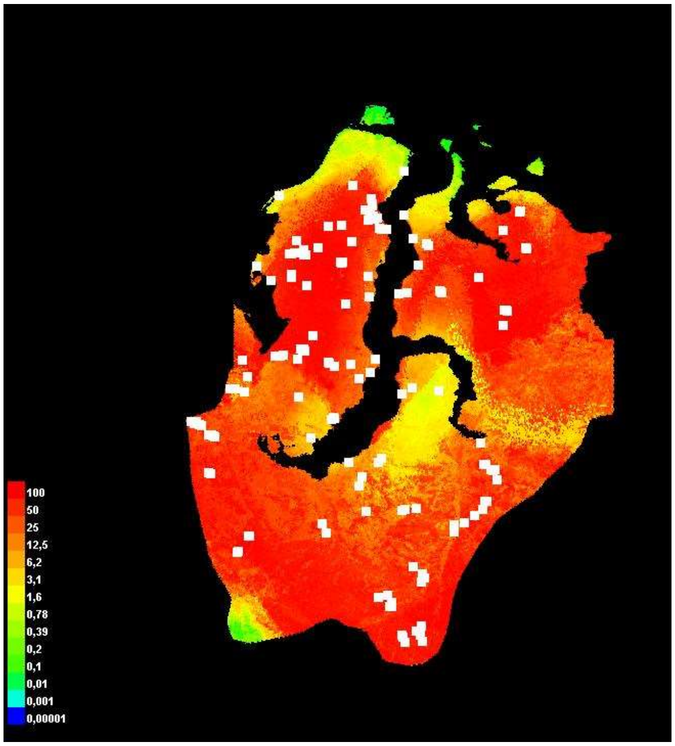

Arctous alpina

Full set

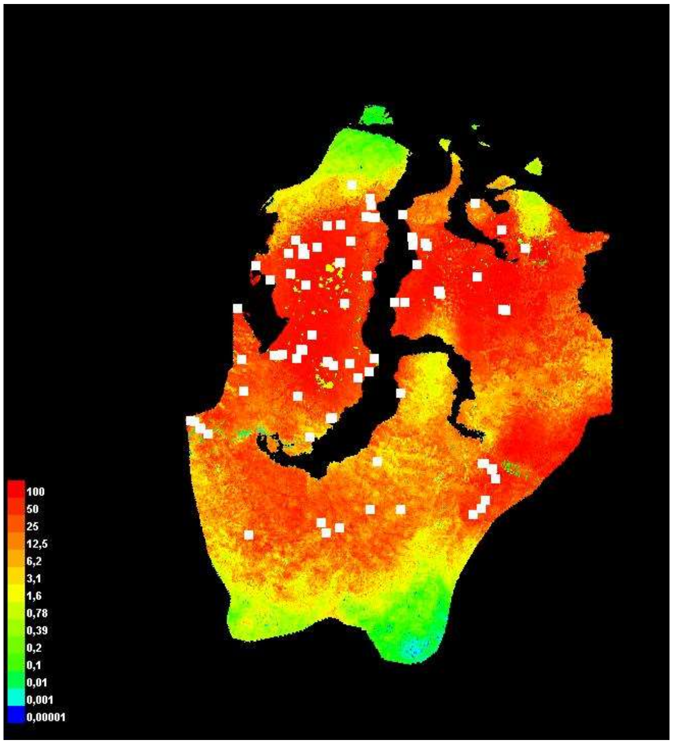

Subset 1 (Automatic)

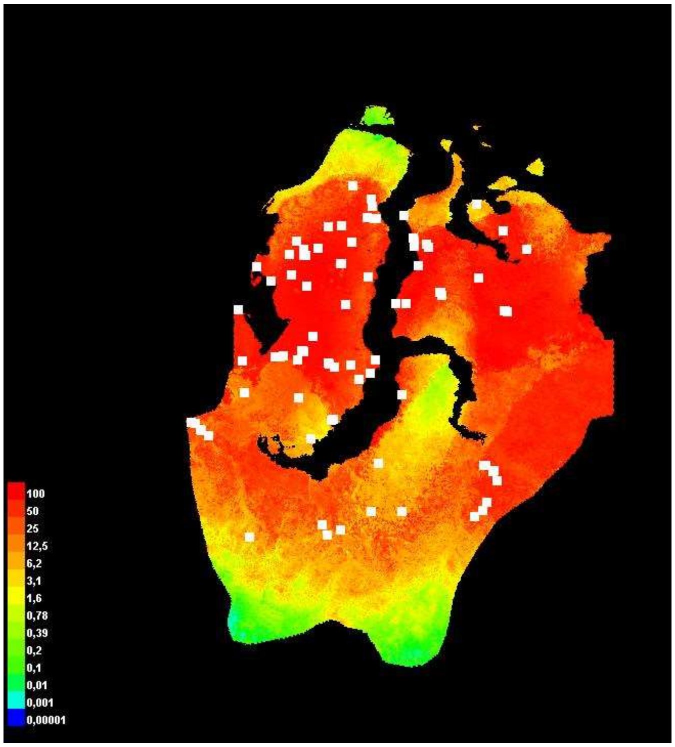

Subset 2 (PCA)

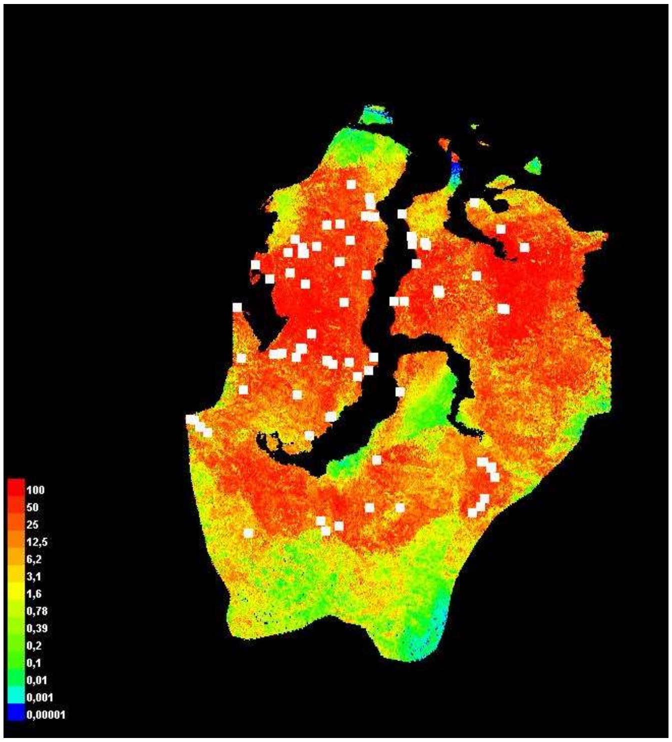

Subset 3 (Expert)

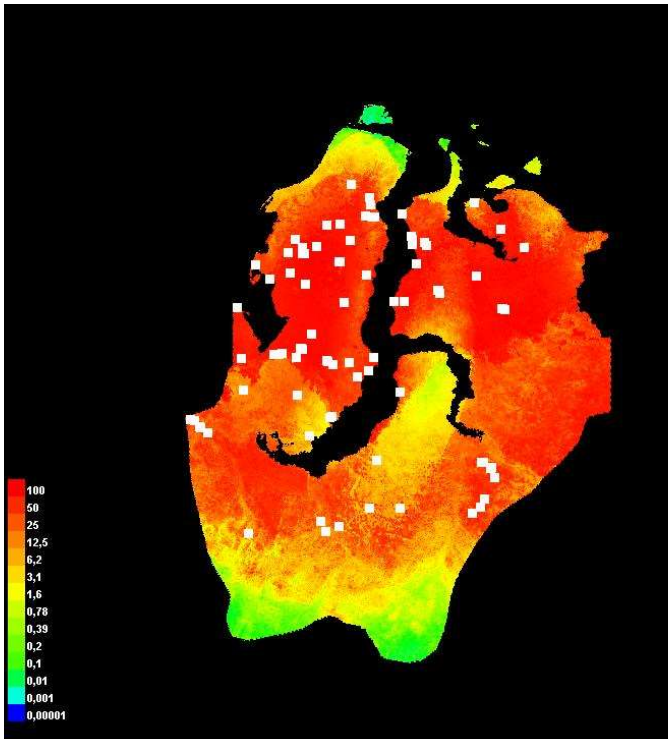

Supplement: Supplementary file 4 — Appendix S4 [file ECE3-13-e10545-s004.pdf]
