# Supplementary material for: The High–Low Arctic boundary: How is it determined and where is it located?
Source: Ecol Evol. 2023 Sep 28;13(10):e10545. doi: 10.1002/ece3.10545 (PMC10539046; doi:10.1002/ece3.10545)

Appendix 5. Biodiversity analysis.

Diversity of vascular, moss and lichen species depending on the distance from the High-Low Arctic boundary is shown below. Numbers along the x axis indicate the range of distances from the boundary for plots within each box, with negative numbers indicating sites north of the boundary and positive number sites south of the boundary. Plots shown in the (-30, 30) box plot are all within 30 km of the boundary and have the highest species richness.

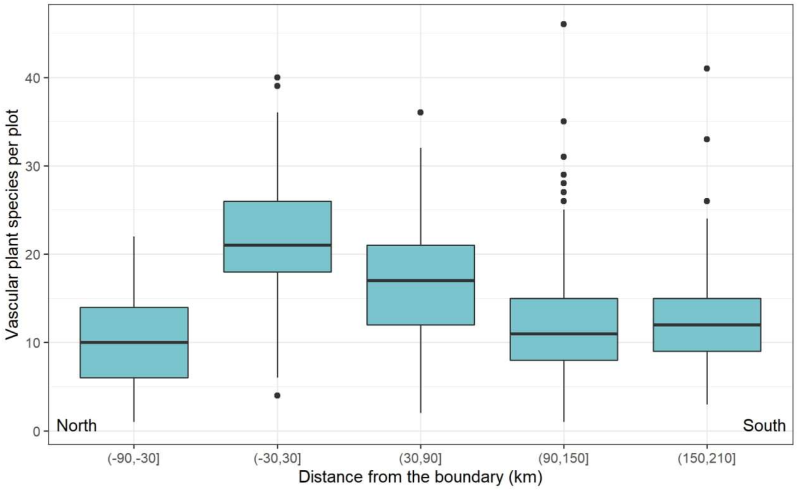

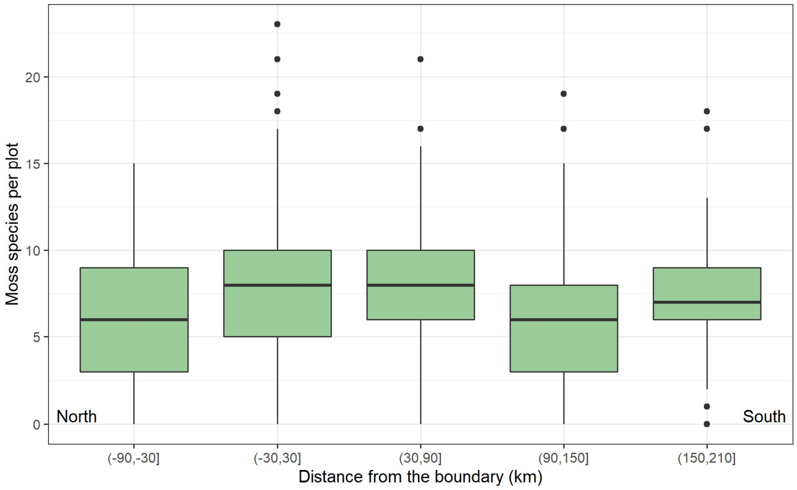

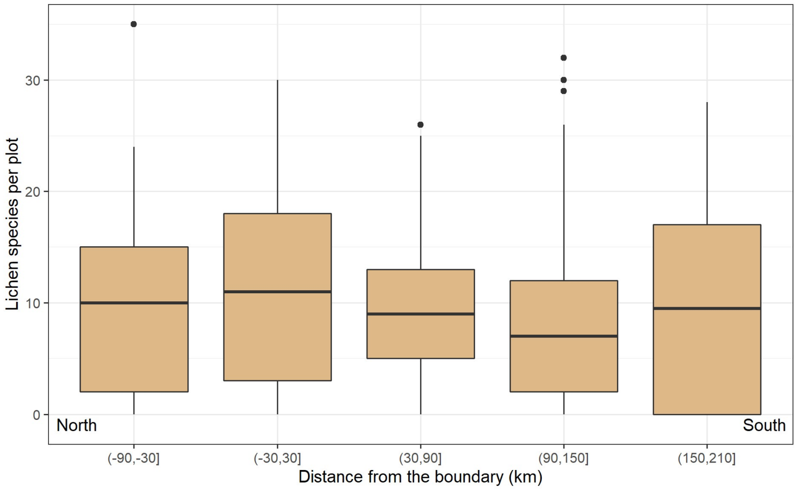

Supplement: Supplementary file 5 — Appendix S5 [file ECE3-13-e10545-s003.pdf]
